# Parasitological efficacy of sulfadoxine-pyrimethamine and amodiaquine for seasonal malaria chemoprevention in Africa

**DOI:** 10.1038/s41467-026-78189-2

**Published:** 2026-10-01

**Authors:** Chayanin Sararat, Paul Sondo, Craig Bonnington, Jane Achan, Chukwudi A. Nnaji, Anna Y. Burkhard, Gauthier Tougri, Adama Traore, Aimé Parfait Korou Yaro, Anthony Nuwa, Richard Kajubi, Kanokon Suwannasin, Stephane Proux, Urairat Koesukwiwat, Sonia Enosse, Ivan Alejandro Pulido Tarquino, Stije J. Leopold, Arjen M. Dondorp, Halidou Tinto, James K. Tibenderana, Joel Tarning, Mallika Imwong, Francois H. Nosten, James A. Watson, Nicholas J. White

**Affiliations:** 1 Mahidol-Oxford Tropical Medicine Research Unit, Faculty of Tropical Medicine, Mahidol University, Bangkok, Thailand; 2 Centre for Tropical Medicine and Global Health, Nuffield Department of Medicine, University of Oxford, Oxford, UK; 3 Infectious Diseases Data Observatory, Oxford, UK; 4 Institut de Recherche en Sciences de la Santé- Clinical Research Unit of Nanoro (CRUN), Nanoro, Burkina Faso; 5 Malaria Consortium, London, UK; 6 Programme National de Lutte contre le Paludisme, Ouagadougou, Burkina Faso; 7 Malaria Consortium, Ouagadougou, Burkina Faso; 8 Malaria Consortium, Kampala, Uganda; 9 Shoklo Malaria Research Unit (SMRU), Mahidol Oxford Tropical Medicine Research Unit, Mae Sot, Thailand; 10 Malaria Consortium, Maputo, Mozambique; 11 Division of Clinical Pharmacology, Department of Laboratory Medicine, Karolinska Institutet, Stockholm, Sweden; 12 Department of Molecular Tropical Medicine and Genetics, Faculty of Tropical Medicine, Mahidol University, Bangkok, Thailand

**Keywords:** Malaria, Epidemiology, Paediatric research, Clinical trials

## Abstract

Seasonal malaria chemoprevention (SMC) with sulfadoxine-pyrimethamine plus amodiaquine (SPAQ) protects millions of African children, yet the contribution of each drug to preventive efficacy remains unclear. We pooled individual-level data on drug concentrations and malaria infection in 934 children from Uganda (NCT05323721), Mozambique (NCT05186363), and Burkina Faso (NCT05478954). The primary outcome was malaria infection by day 28, and the secondary outcome was the day 28 qPCR cycle threshold value. From natural variation in drug exposure, desethylamodiaquine, the active amodiaquine metabolite, was the primary determinant of malaria infection (OR 0.55, 95% CI 0.44–0.68 per standard deviation increase in log concentration). Children with low day 28 desethylamodiaquine exposure (<24 ng/mL) had nearly twice the risk of malaria infection (RR 1.9, 95% CI 1.5–2.4). Sulfadoxine and pyrimethamine showed only weak associations, consistent across countries despite varying resistance haplotypes. SPAQ efficacy appears primarily amodiaquine-driven and the role of single-dose sulfadoxine–pyrimethamine merits further evaluation.

## Abstract (web summary)

In this study, the authors show that post-treatment desethylamodiaquine concentration is the primary pharmacokinetic determinant of protection against malaria infection in African children who are given sulfadoxine-pyrimethamine and amodiaquine for seasonal malaria chemoprevention, with little apparent effect from sulfadoxine-pyrimethamine.

## Introduction

Malaria remains the leading cause of preventable death in young children in sub-Saharan Africa. In parts of sub-Saharan Africa such as the Sahel region, malaria transmission is highly seasonal with most cases concentrated in 4–5 months of the year during the rainy season. Seasonal malaria chemoprevention (SMC) consists of giving monthly treatment doses of slowly eliminated antimalarials to young children to prevent infection and illness during the peak risk period^1^. SMC has been recommended by the World Health Organisation (WHO) since 2012^2^ and in 2025, over 50 million children received SMC. In 2022, WHO broadened their recommendation around SMC allowing countries to choose how and where to implement it^3^. SMC can now be used in a wider age group, in areas where there is high grade resistance to sulfadoxine-pyrimethamine, and over more cycles each year. There is little evidence to support this broader recommendation.

The drug combination currently recommended by WHO for SMC is sulfadoxine-pyrimethamine plus amodiaquine (SPAQ)^3^. Sulfadoxine-pyrimethamine (SP) has been used as an antimalarial for over 50 years; a single treatment dose reliably prevented recurrence of infection with sensitive parasites within one month, although its efficacy is now compromised by multiple mutations in the *pfdhfr* gene (conferring resistance to pyrimethamine) and the *pfdhps* gene (conferring resistance to sulfadoxine)^4^. Nearly all *P. falciparum* parasites in West Africa are “quadruple mutants”, characterised by the *pfdhps* 437G mutation but lacking the *pfdhps* 540E mutation. However, in East and Southern Africa, nearly all *P. falciparum* parasites are “quintuple mutants” (540E)^5^ or sextuple mutants, with substantially less susceptibility to sulfadoxine. The other component of SMC, amodiaquine, is rapidly converted to its active metabolite desethylamodiaquine which has a relatively long elimination half-life (approximately 10 days)^6^. Amodiaquine is given over three days to obtain sufficiently high drug concentrations for one month. For SMC to work effectively, children must maintain adequate drug concentrations in the blood throughout the protection window. Low drug exposure results in reduced protection^7^.

To better characterise the relationship between drug exposure and protection against malaria infection, in 2022 Malaria Consortium conducted three Chemoprevention Pharmacokinetic-Efficacy Studies (CPES) of SPAQ in Uganda^8^, Mozambique^9^, and Burkina Faso. These studies were embedded in larger implementation studies of SMC. The studies enroled children under 5 years who were eligible for SMC; the children were administered their first cycle of SPAQ and then were followed up for one month with dried blood spots taken at baseline and on days 7 and 28 after start of treatment to assess drug exposure and malaria infection by PCR. This approach allows for characterisation of the relationship between drug levels and protective efficacy against blood parasitaemia. In this pooled analysis, we look at the determinants of parasitological efficacy for SPAQ and assess whether current dosing regimens of sulfadoxine, pyrimethamine, and amodiaquine administered during standard SMC campaigns provide adequate protection over one month against *Plasmodium* infections in children under 5. If protection is inadequate, an important question is whether this is due to underdosing or drug resistance. This will contribute to understanding whether the dosage or choice of drugs needs to be modified to achieve the full potential for SMC to prevent malaria infection in the future.

## Results

### Participant characteristics

**Fig. 1:**
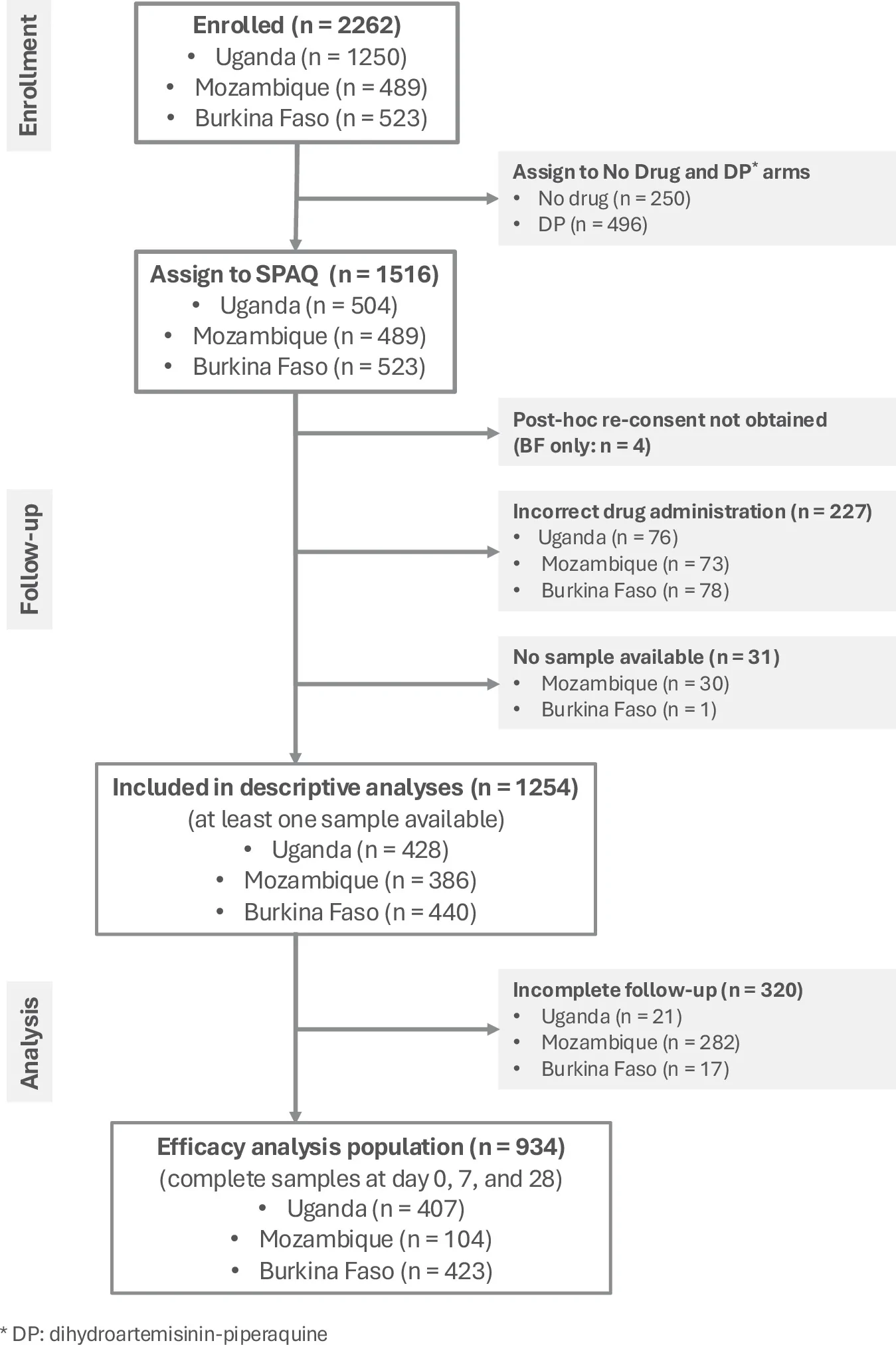
Study fl ow diagram. Flow of participants through enrolment, exclusion, follow-up, and inclusion in the analysis population across the three study sites.

A total of 1516 children were enroled into SPAQ arms across the three studies: Uganda (*n* = 504; 11 July 2022–23 August 2022), Mozambique (*n* = 489; 18 January 2022–30 January 2022), and Burkina Faso (*n* = 523; 10 July 2022–11 August 2022) (Fig. 1). After excluding children with drug concentrations suggesting possible additional or off-schedule dosing, no biological samples for laboratory evaluation, or lacking post-hoc re-consent (Burkina Faso), 934 children were included in the analysis population (Fig. 1 and Table 1). We restricted the analysis population to children with blood samples available at all three time points (days 0, 7 and 28) in order to have a fair comparison across the three drugs which have different pharmacokinetic profiles. The proportion of complete follow-up varied substantially by site. Only 104 children (21%) in Mozambique had complete sampling, compared with 81% in Uganda and 81% in Burkina Faso. Overall, participants were broadly comparable across sites with respect to age, sex, and nutritional status. However, the participants from Uganda tended to be younger and showed higher levels of malnutrition, particularly when assessed using mid-upper arm circumference-for-age (z-MUAC; Table 1 and Fig. S1).

**Table 1:** Demographic and anthropometric characteristics of participants in the analysis population by country. For continuous variables we give the median (IQR) values

Site	Uganda	Mozambique	Burkina Faso
Enroled participants (*n*)	504	489	523
Analysis population (*n*, %)	407 (81%)	104 (21%)	423 (81%)
Age (months)	33 (12–47)	33 (21–46)	32 (18–47)
Male sex (%)	212 (52%)	53 (51%)	223 (53%)
Weight (kg)	11.4 (9.0–14.0)	11.3 (8.6–15.0)	11.4 (9.1–13.5)
z-weight	−0.8 (-1.7 to 0.0)	−1.1 (−2.9 to 0.3)	−1.2 (−1.8 to −0.4)
MUAC (cm)	13 (12–14)	15 (14–16)	15 (14–16)
z-MUAC	−2.1 (−2.9 to −1.2)	−0.1 (−0.8 to 0.6)	−0.6 (−1.1 to 0.0)
Malaria infection at baseline (*n*, %)	239 (59%)	68 (65%)	171 (40%)

### Malaria infection prevalence

**Fig. 2:**
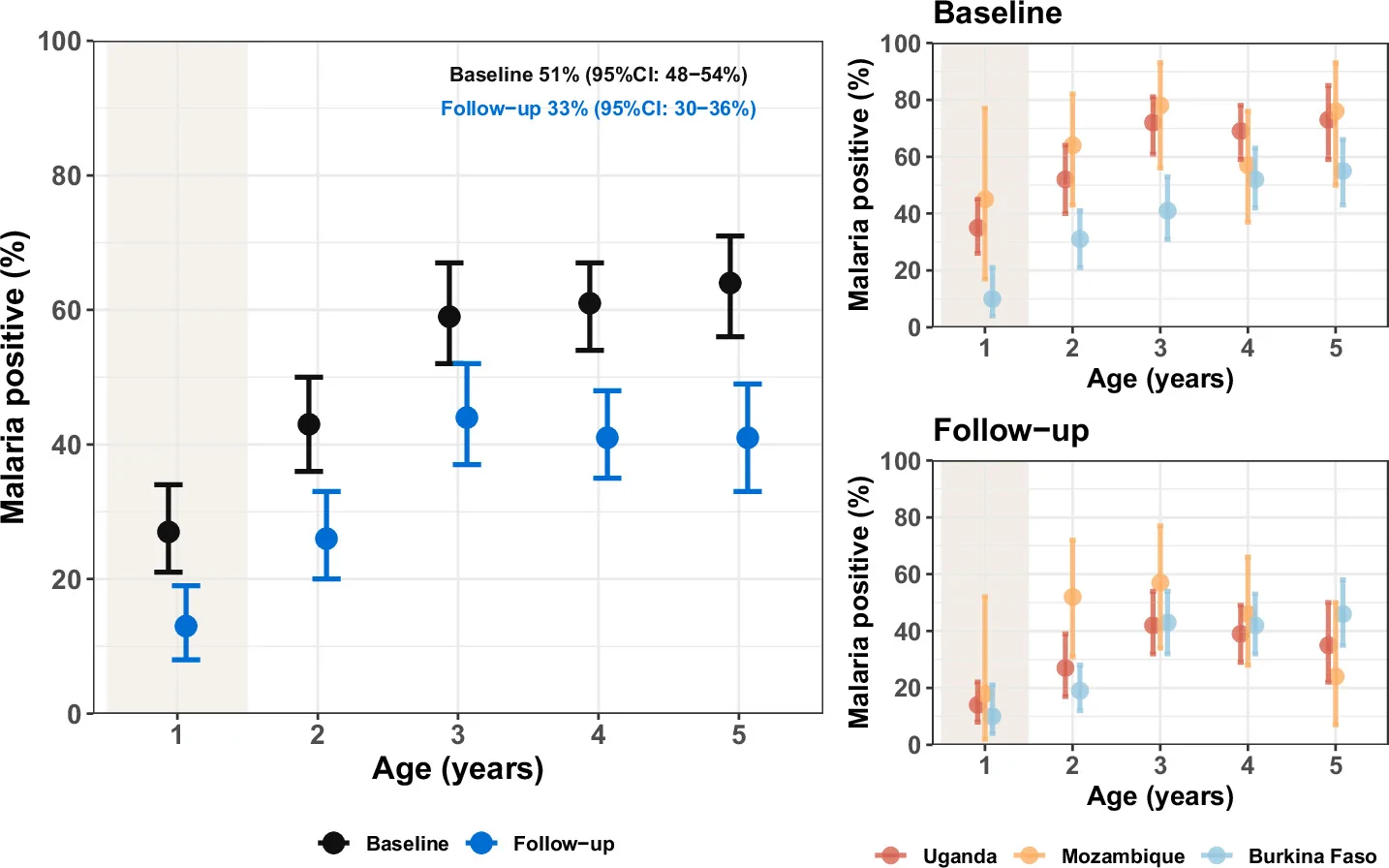
Malaria positivity at baseline and day 28 of follow-up period (*n* = 934). Malaria infection was defined as either PCR positivity at day 28 or intercurrent clinical malaria. Children under one year of age received half the total dose of SPAQ relative to those aged 1–5 (shaded area). Prevalence point estimates (points) are shown with 95% binomial confidence intervals (error bars).

Half of the children in the analysis population (478/934) had qPCR-detectable malaria infections at baseline. The proportion of children infected at baseline was lower in Burkina Faso (40%), likely due to the timing of the first SMC dose relative to the start of the transmission season. Infection prevalence increased with age, with a higher proportion of older children harbouring detectable infections (Fig. 2 and Figure S2). Most infections were caused by *Plasmodium falciparum*, which was detected in 91% (435/478) of infected children overall. Among PCR-positive samples, the proportion attributable to *P. falciparum* was similar across sites: 223/239 (93%) in Uganda, 63/68 (93%) in Mozambique, and 149/171 (87%) in Burkina Faso (Table S1 and Figure S3). Estimates of overall and *P. falciparum* prevalence differed only slightly when calculated using all available baseline samples (Table S1).

28 days after SMC administration, 33% (310/934) of children had a malaria infection (primary analysis outcome, defined as either qPCR positive on day 28 or intercurrent malaria) (Table 2). Of these 310 children, 44 children had experienced intercurrent clinical malaria (24 (6%) in Uganda, 3 (3%) in Mozambique, and 17 (4%) in Burkina Faso). *P. falciparum* remained the predominant species at day 28, detected in 238/284 (84%) of PCR-positive children, corresponding to 78% of infections in Uganda, 93% in Mozambique, and 86% in Burkina Faso.

**Table 2:** Summary of efficacy outcomes by country

Site	Uganda	Mozambique	Burkina Faso
Malaria infection (*n*, %)*	125/407 (31%)	45/104 (43%)	140/423 (33%)
PCR positive on day 28 (*n*, %)	109/407 (27%)	44/104 (42%)	131/423 (31%)
Intercurrent clinical malaria^*^ (*n*, %)	24/407 (6%)	3/104 (3%)	17/423 (4%)
**Cycle threshold (Ct) values**			
*N* (Ct = 40)	298	60	292
*N* (Ct <40)	109	44	131
Range (min-max)^a^	18.7–38.6	19.8–37.9	18.8–38.9
Mean^a^	33.8	32.6	31.9
Median (IQR)^a^	35 (32.5–36.1)	34 (31.2–35.9)	34 (29.5–35.4)

*Malaria infection is defined as PCR-positive by day 28 or intercurrent malaria illness (fever or history of fever within 24 hours with a positive RDT). ^a^In samples with Ct <40 only.

A substantial burden of *P. ovale* infections were observed, detected in 86/478 (18%) of baseline infections and 29/284 (10%) by day 28 (Table S1 and Fig. S3). Of the 86 *P. ovale* infections detected at baseline, 43 occurred mixed with *P. falciparum. P. malariae* was detected infrequently (11 patients, 2%), with 10 cases in Uganda, and one in Burkina Faso, and was almost entirely cleared by day 28, with only a single case in Uganda.

### SMC drug concentration profiles

**Fig. 3:**
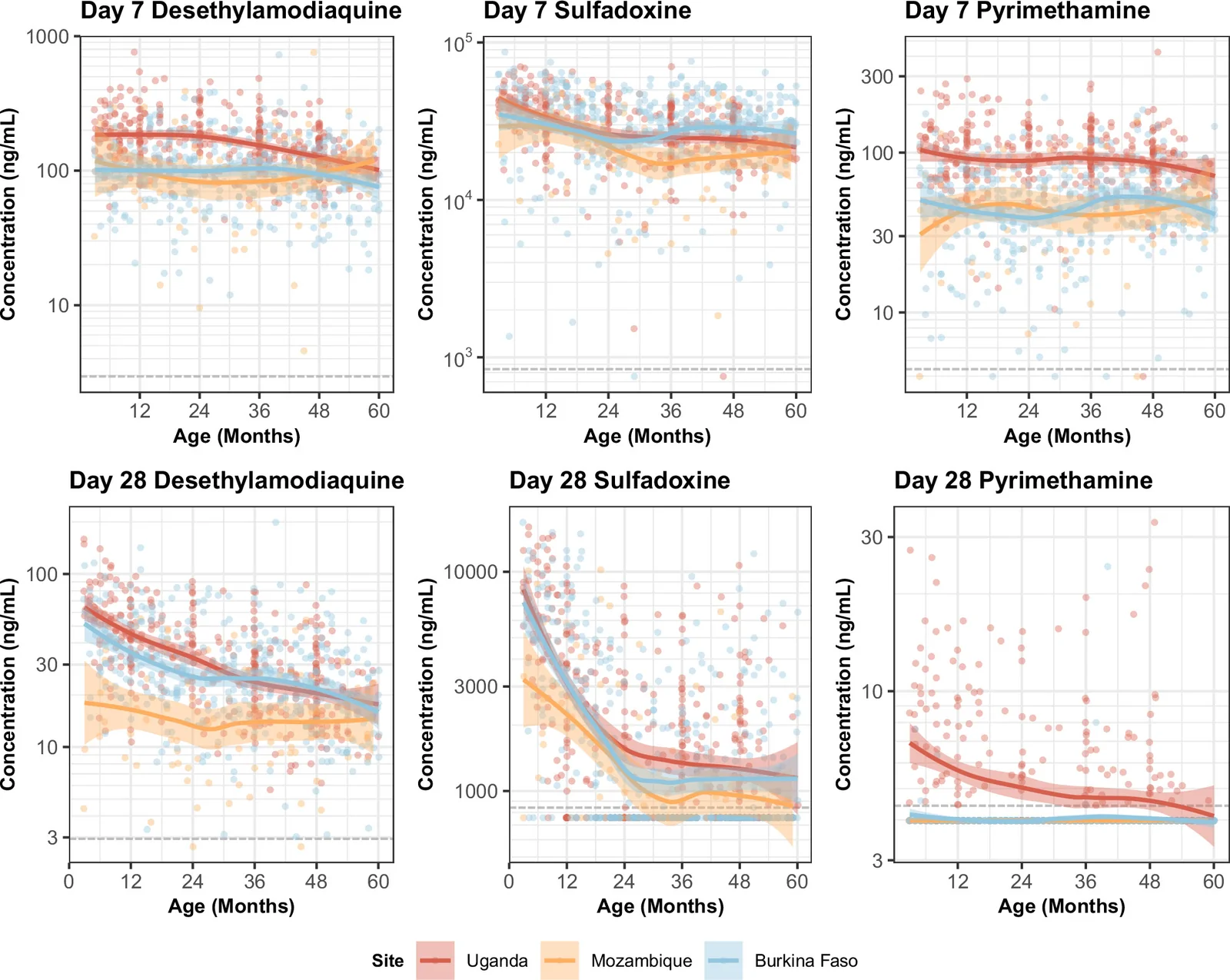
Drug concentration profiles of desethylamodiaquine, sulfadoxine, and pyrimethamine at day 7 and day 28 post-treatment, as a function of age in years with added jitter, coloured by study site. Lines show spline trends (mean) with shaded areas representing 95% confidence intervals. Horizontal dashed lines denote the lower limit of quantification (LLOQ) for each drug.

The concentrations of sulfadoxine, pyrimethamine, desethylamodiaquine, and amodiaquine were measured on days 7 and 28. Day 7 concentrations showed relatively limited variability and were evenly distributed across age groups. In contrast, day 28 concentrations declined with increasing age, most likely reflecting slower clearance in young children. Age-related effects were most apparent for sulfadoxine and desethylamodiaquine (Fig. 3). For sulfadoxine, the median day 28 concentration among children under 1 year was 5045 ng/ml with undetectable concentrations in 8% of children, whereas the median sulfadoxine level was undetectable in 50% of children above 1 year. Across sites, sulfadoxine levels were broadly comparable, whereas pyrimethamine concentrations were almost twice as high in Uganda (Fig. 3, Table S2). Amodiaquine concentrations fell below the threshold for reliable laboratory quantification (1.87 ng/mL) in 42% of children by day 7 and 94% by day 28, consistent with its rapid elimination half-life^10^. In contrast, for its active metabolite, desethylamodiaquine, levels were highest in Uganda at day 7. By day 28, desethylamodiaquine levels were similar in Uganda and Burkina Faso, while remaining lower in Mozambique.

### Drug exposure and protection against malaria

**Fig. 4:**
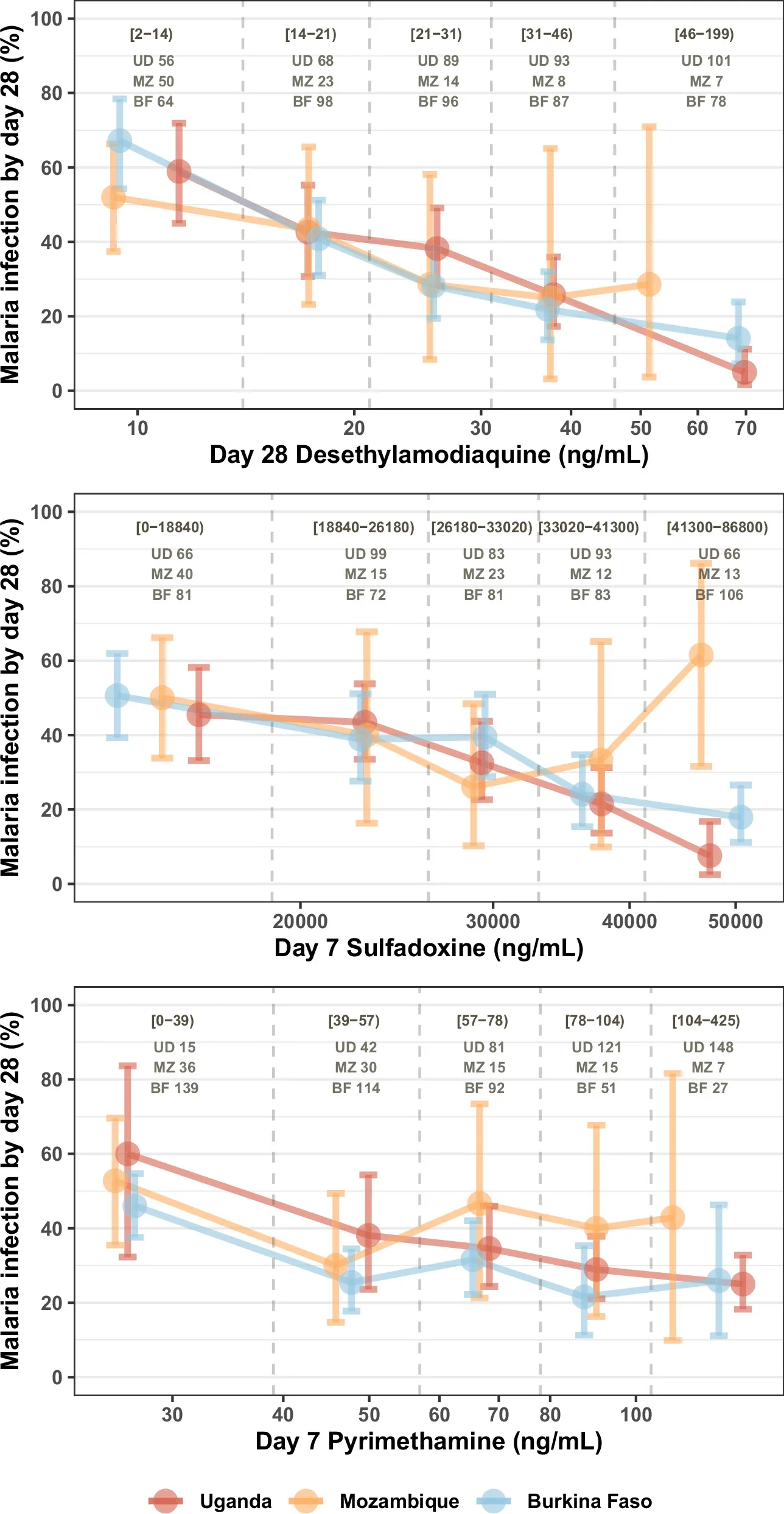
Relationship between drug concentrations and malaria infection by day 28 (*n* = 934). Participants were ranked by the drug concentration and divided into quintiles. For each site and quintile, the proportion of malaria-positive children (PCR+ or intercurrent clinical malaria) is shown as points with 95% exact binomial confidence intervals (error bars).

Across all three main SMC drug exposures (sulfadoxine, pyrimethamine, and desethylamodiaquine), higher post-treatment concentrations were generally associated with lower risk of malaria by day 28 (Fig. 4). Although breakthrough infections occurred among some children with high drug exposure, parasite densities in these cases were lower than among children with lower drug exposure (Fig. S4). Drug concentrations among children who experienced intercurrent clinical malaria during follow-up are summarised in Fig. S5. We compared four exposure metrics for each drug: the day 7 concentration, day 28 concentration, AUC from day 7 to day 28, and terminal elimination half-life (Fig. S6). For desethylamodiaquine, the day 28 concentration was the best predictor of malaria infection; for sulfadoxine, it was the day 7 concentration. For pyrimethamine, only the day 7 concentration was evaluable, as day 28 concentrations were below the LLOQ in 83% of children overall (64% in Uganda, 100% in Mozambique, and 99% in Burkina Faso).

Under a multivariable model including these three selected drug exposures, desethylamodiaquine exposure showed the strongest association with malaria infection by day 28, after adjustment for site, calendar time, age, and malaria infection at baseline. There was weaker evidence of an association with sulfadoxine exposure, and no clear protective effect was observed for the pyrimethamine exposure (Fig. S7). For the desethylamodiaquine exposure, the relationship with malaria infection on day 28 was approximately linear on the logit scale (best fit in a generalised additive model). After standardising each drug exposure so the effects were comparable, one standard deviation increase in the log desethylamodiaquine exposure was associated with an odds ratio for malaria infection by day 28 of 0.55 (95% CI 0.44−0.68) (Fig. S7). The corresponding odds ratios for a one-standard-deviation increase in the log sulfadoxine and pyrimethamine concentrations were 0.80 (95% CI 0.63–1.00) and 0.88 (95% CI 0.67–1.15), respectively.

We further evaluated whether the protective effect of drug exposure varied by age. Interaction models were fitted separately for each drug. There was no clear evidence that the associations for desethylamodiaquine or pyrimethamine differed across the age range of enroled children. In contrast, younger children appeared to derive greater benefit from higher sulfadoxine exposure, with the protective effect diminishing with increasing age. The interaction between age (in years) and day 7 sulfadoxine concentration corresponded to an approximately 20% attenuation in the protective odds ratio per additional year of age (interaction coefficient = 0.18 on the log-odds scale, *p* = 0.0007). Despite this age-dependent effect, the overall conclusions regarding the dominant role of desethylamodiaquine exposure remained unchanged.

The predicted probability of malaria infection by day 28 declined markedly with increasing day 28 desethylamodiaquine concentrations (Fig. S8). The risk fell to approximately below 40% once concentrations exceeded the population geometric mean (z-score = 0; corresponding to approximately 24 ng/mL). Notably, fewer than 25% of children in Mozambique achieved concentrations above this threshold, compared with more than 50% of children in Uganda and Burkina Faso (Fig. S8).

To estimate the causal effect of low (<24 ng/mL: population mean exposure) versus high day 28 desethylamodiaquine exposure, we used inverse probability weighting based on the known confounders: age, nutritional status, sex, and site, allowing for an unbiased comparison of low versus high exposure (Fig. S9, Fig. S10). Age was the primary determinant of low day 28 desethylamodiaquine exposure (Fig. S11). Children with low day 28 desethylamodiaquine exposure had a risk ratio for malaria infection by day 28 of 1.9 (95% CI: 1.5–2.4). In Uganda, 63% (255/407) had day 28 desethylamodiaquine >24 ng/mL, whereas in Burkina Faso this was 55% (232/423) and in Mozambique 24% (25/104).

**Fig. 5:**
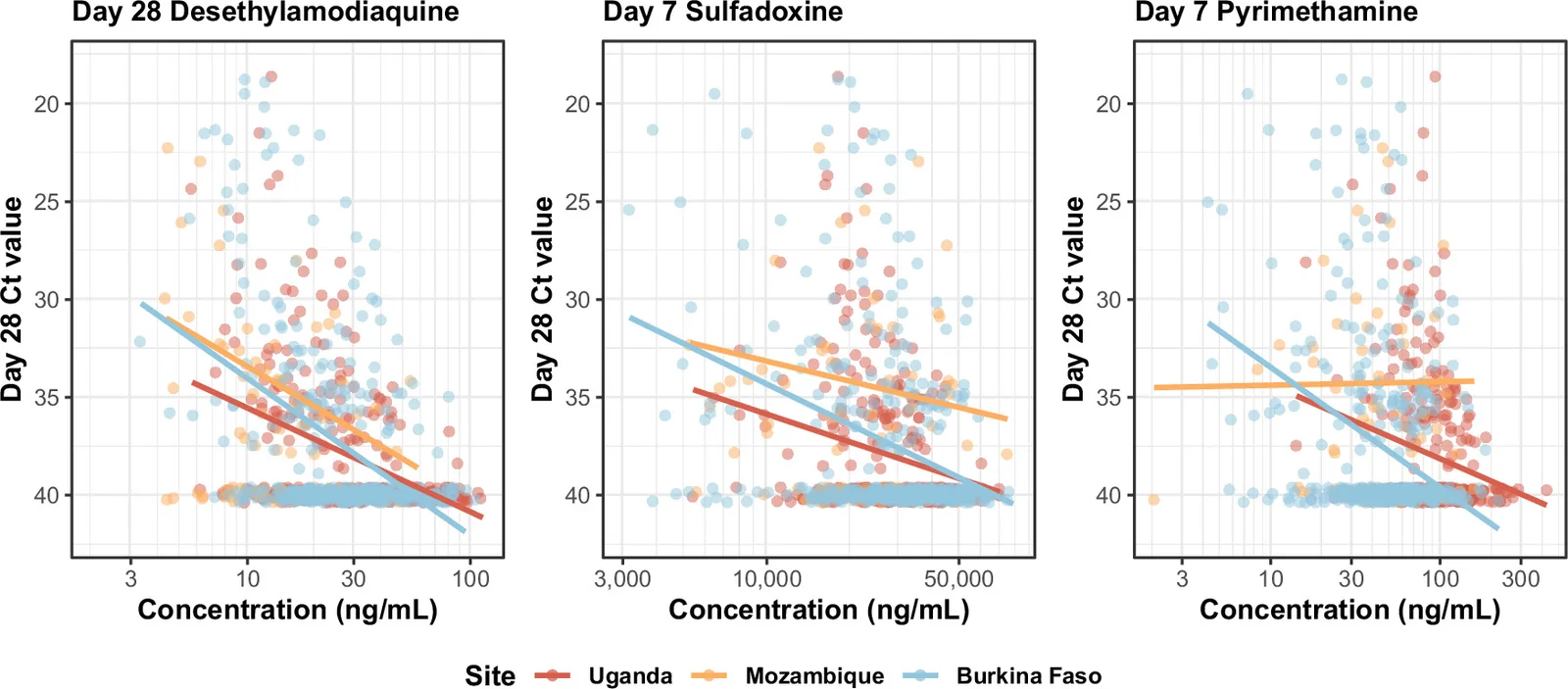
Relationships between *Plasmodium spp*. Ct values and drug concentrations. Scatter plots show observed D28 Ct values in relation to D28 desethylamodiaquine, D7 sulfadoxine and D7 pyrimethamine concentrations. Ct values were analysed as left-censored at a Ct value of 40. Solid lines represent estimated trends from Tobit regression models, adjusting for age, study site, baseline Ct value, mid-upper arm circumference, and enrolment date.

Higher day 28 desethylamodiaquine concentrations were associated with lower parasitaemia on day 28, as reflected by higher Ct values (Fig. 5). Associations between day 28 Ct values and day 7 sulfadoxine and pyrimethamine concentrations were weaker. In left-censored Tobit models accounting for Ct values at the limit of quantification, one standard deviation increase in desethylamodiaquine concentrations was associated with a 2 Ct value increase in day 28 (equivalent to an approximately 4-fold reduction in parasite density, *p* < 0.001). Each standard deviation increase in sulfadoxine concentration attenuated the desethylamodiaquine effect by approximately 1 Ct (approximately 2-fold reduction in the parasite density reduction, *p* < 0.001, site-specific analyses are presented in Supplementary Results). Baseline Ct was independently associated with day 28 Ct, whereas age and nutritional status were not. Significant concentration-dependent attenuation of the desethylamodiaquine protective effect was observed at higher sulfadoxine and pyrimethamine concentrations (site-specific analyses are presented in Supplementary Results), suggesting diminishing incremental effects of higher desethylamodiaquine concentrations at higher levels of partner drug exposure (saturation of effect, Fig. S12).

### Molecular resistance markers

**Fig. 6:**
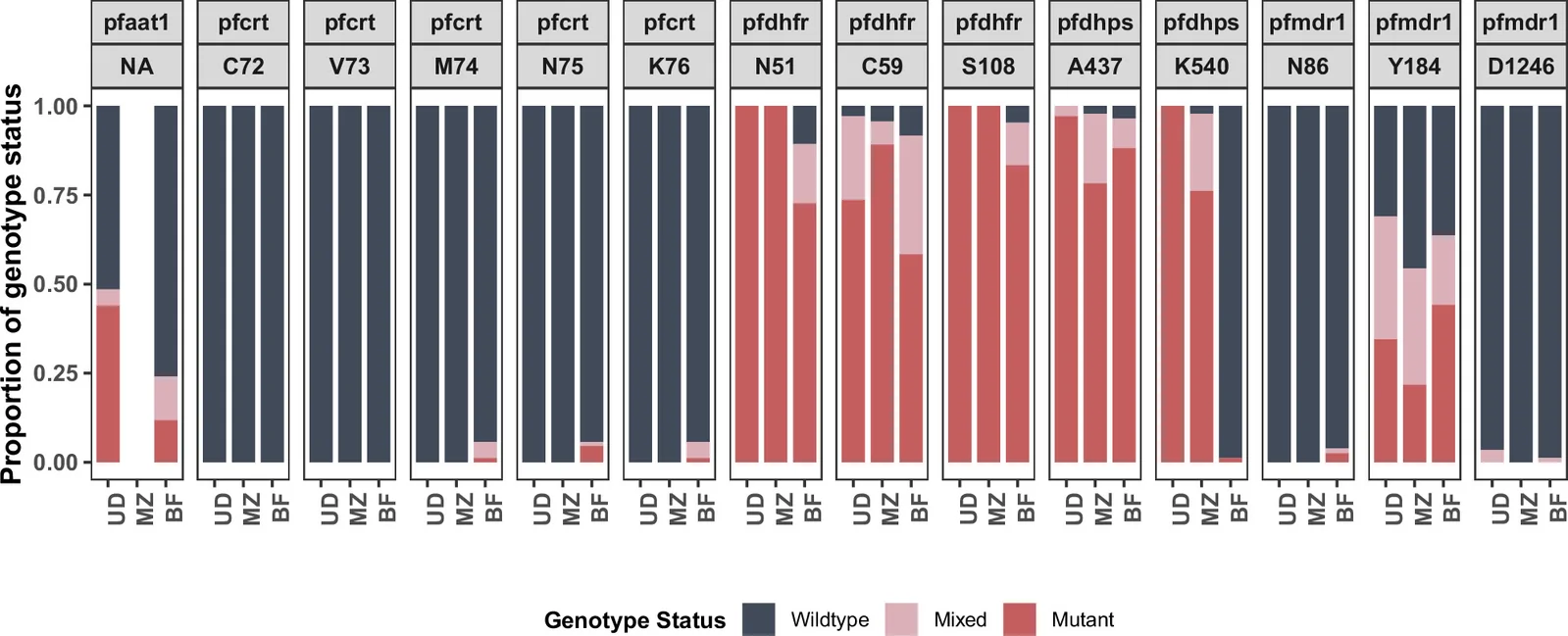
Proportion of key SPAQ-resistance–associated mutations at baseline across study sites. Stacked bars coloured by genotype status (pure wild-type, mixed mutant, and pure mutant). Markers include key positions in *pfaat1*, *pfcrt*, *pfdhfr*, *pfdhps*, and *pfmdr1*.

Known molecular markers associated with SP and AQ resistance were genotyped in samples collected at baseline (Fig. 6, Table S3, and Fig. S13). Mutations in *pfdhps* (A437G, K540E) and *pfdhfr* (N51I, C59R, S108N) associated with reduced sulfadoxine and pyrimethamine susceptibility, respectively^11,12^, were highly prevalent across sites, predominantly as pure mutant genotypes. The *pfdhps* 437G mutation was fixed or near-fixed in all settings: Uganda 97% pure + 3% mixed, Mozambique 78% + 20%, and Burkina Faso 88% + 8%, respectively. At *pfdhps* 540, Uganda and Mozambique had high frequencies of the pure mutant 540E allele, whereas Burkina Faso remained almost entirely pure wild-type, with rare mixed infections.

The *pfdhfr* mutations were also widespread. The 51I and 108N alleles were fixed as pure mutants in Uganda and Mozambique, and predominant in Burkina Faso (73% and 83%, respectively), where a small proportion of mixed infections was detected. The 59R mutation occurred at high prevalence, 91% in Burkina Faso and 98% in Uganda, with mixed genotypes more common at these sites (24% and 33%, respectively). In Mozambique, most 59R infections were pure mutants (89%), with only a minority mixed (7%).

For AQ-resistance–associated markers, we genotyped *pfcrt* codons 72–76^13^ and *pfmdr1* codons 86, 184, and 1246^11^. Nearly all samples carried the wild-type pfcrt CVMNK haplotype at codons 72–76, with only a small number of mixed or mutant haplotypes observed in Burkina Faso. *pfmdr1* mutations were generally uncommon. At codon 86, the wild-type N allele predominated at all sites, with only rare mixed or pure 86Y mutants found in Burkina Faso. Similarly, at codon 1246, both pure and mixed mutant infections were only sporadically observed in Uganda and Burkina Faso. The 184 F allele occurred at moderate frequencies across sites, appearing mainly as pure mutant or mixed 184 F/Y genotypes.

## Discussion

Effective malaria chemoprevention relies on sustaining sufficiently high drug exposures throughout the at-risk period to prevent reinfection and illness. Current SMC deployment consists of monthly administration of two drugs, sulfadoxine-pyrimethamine given as a single dose, and amodiaquine given once a day over three days. The initial randomised trials of SMC did not determine the relative contribution of each drug to the overall protective efficacy. Knowing the relative contribution of amodiaquine is important because it determines the relationship between adherence to the full three-day course and efficacy. This pharmacometric assessment of SMC uses natural variation in drug exposures due to variation in body weight, absorption and elimination to compare the contribution of sulfadoxine-pyrimethamine versus amodiaquine for parasitological efficacy. We show that the day 28 desethylamodiaquine concentration was the primary determinant of parasitological efficacy. Sulfadoxine (the slower-eliminated component of sulfadoxine-pyrimethamine) contributed little to the protection against malaria infection. A notable and somewhat surprising result was the consistency of the exposure–response relationships across the three study sites. This is particularly striking given the different known sulfadoxine-pyrimethamine resistance haplotypes across the three settings. The consistency across sites strengthens the conclusion that amodiaquine exposure is the main determinant of parasitological efficacy in SMC.

Sulfadoxine-pyrimethamine was once widely used for the treatment of malaria in Africa in the 1990s and early 2000s but rapidly succumbed to resistance. In Africa, sulfadoxine-pyrimethamine is now only used for intermittent preventative treatment. Resistance to sulfadoxine-pyrimethamine is thought to be generally worse in East and Southern Africa where the *pfdhps* K540E mutation (“quintuple mutant”) is highly prevalent. However, parasites in West Africa are also partially resistant to sulfadoxine-pyrimethamine, with a very high prevalence of the *pfdhps* A437G mutation (“quadruple mutant”). Data from treatment studies done over 20 years ago suggest that the duration of protection from recurrent infection with quadruple mutants is ~30 days, compared with ~16 days for the quintuple mutant^14^. This would suggest that sulfadoxine should have a much greater contribution in Burkina Faso compared to Uganda and Mozambique. The data from these studies do not support this prediction and show little contribution of the sulfadoxine component in West or in East and Southern Africa in terms of parasitological efficacy. Prevention of infection at day 28 was only observed in children who had high desethylamodiaquine levels (e.g. >24 ng/ml: population geometric mean exposure). Exploratory site-specific analyses suggested slightly stronger sulfadoxine protective effect in Burkina Faso on day 28 parasitaemia, compared to Uganda and Mozambique, but this was not significant. This implies that adherence to the three-day course of amodiaquine is essential for the effectiveness of SMC as currently deployed.

Amodiaquine is an old antimalarial drug that was previously used extensively for prophylaxis in travellers because it retained efficacy against chloroquine-resistant *P. falciparum*. However, its use in prophylaxis was discontinued in the 1980s because of rare toxicity, notably agranulocytosis and liver injury, in some cases fatal^15^. These effects appear to follow prolonged exposure (weekly or daily prophylaxis) and have not been observed in either treatment or seasonal malaria chemoprevention^16^. However, the dose given in SMC is likely to be the maximum tolerated dose^17^. It could be dangerous to increase the daily dose to obtain higher day 28 concentrations. Given that desethylamodiaquine concentrations are below optimal for many children even after fully observed treatment, this implies that amodiaquine alone cannot achieve very high protective efficacy against malaria infection, defined as PCR-detectable parasitaemia or intercurrent clinical malaria. This supports additional interventions such as seasonal vaccination^18^.

The variation in the sulfadoxine-pyrimethamine ratios across sites, whereby sulfadoxine concentrations were broadly comparable, but pyrimethamine concentrations were almost twice as high in Uganda, cannot reflect differences in adherence given that both drugs are co-administered as a single tablet. Biological factors, including possible pharmacogenetic differences in drug metabolism, might contribute to this inter-site variation. Sulfadoxine undergoes well-characterised hepatic metabolism and is primarily eliminated by renal filtration^4^, which might account for its consistent concentrations across sites. In contrast, pyrimethamine is metabolised via hepatic pathways that remain incompletely characterised^19^, rendering it potentially more susceptible to inter-population pharmacogenetic variability. For desethylamodiaquine, incomplete adherence to the second and third amodiaquine doses at sites without confirmed directly observed therapy remains the most plausible contributor to inter-site variability. *CYP2C8* polymorphisms, prevalent in West African populations^20^ and shown in vitro to impair amodiaquine metabolism^21^, might represent an additional biological determinant. Prospective CYP genotyping in future SMC pharmacokinetic studies would help disentangle the relative contributions of adherence and pharmacogenetic variation to inter-site differences in drug exposure.

The concentration-dependent attenuation observed between desethylamodiaquine and sulfadoxine (Fig. S12) warrants careful mechanistic interpretation. There does not seem to be a strong pharmacokinetic interaction between amodiaquine and SP^22^. Therefore, the observed saturation in effect is likely to be a result of the pharmacodynamics (near maximal inhibition reached). Further in vitro work better characterizing this interaction as a function of the known SP resistance mutations would be useful to know how these results generalise.

This pharmacometric analysis has some limitations. First, the quality of the data collection and follow-up was variable across three studies. There were substantial issues in data collection and follow-up in Mozambique where complete follow-up data was available in only 21% of participants. Because the exposures of interest are the drug concentrations at day 7 and 28, and the outcome (malaria infection) is measured in the same samples, this is unlikely to introduce bias. Second, it is not possible to entirely differentiate between the causal contribution of higher desethylamodiaquine concentrations versus higher sulfadoxine concentrations. Exposure to both drugs is highly correlated in the same individual (same mg/kg dose; same food effects on absorption and age effects on elimination). The correlation is not perfect, but it is possible that a stronger predictive relationship between desethylamodiaquine and malaria infection compared with sulfadoxine is due to higher accuracy of the desethylamodiaquine measurement (less noise). The effects can only be differentiated fully if the dosage of each drug were randomised independently (e.g. a factorial design). Third, PCR-based detection cannot readily distinguish live from non-viable parasites, as parasite DNA can theoretically persist in blood. Parasite DNA persistence would result in contamination of the PCR-based outcome, thus flattening the exposure-response curve. We think it is highly unlikely that DNA persistence has materially affected our findings for two reasons. The day-28 PCR-estimated parasite densities agree closely with concurrent microscopy-based estimates in samples from Uganda (Fig. S14). In addition, when we use a more stringent Ct value cutoff of 35 cycles (excluding low-density infections or potential false-positive results) we obtain near-identical exposure-response relationships (Supplementary Results). Fourth, the exposure-response relationships inferred in this analysis were derived primarily from qPCR detection of malaria parasites in blood. The primary therapeutic goal of SMC is not to prevent infection but to prevent illness. Parasitological and clinical efficacies are related by definition (illness cannot occur without infection), but the exact relationship has not been characterised. Age is a key effect modifier: any given infection is more likely to become symptomatic in younger children. It could be the case that the clinical efficacy of amodiaquine is higher than its parasitological efficacy if sub-therapeutic concentrations do not prevent infection but sufficiently restrict parasite multiplication, thus preventing symptomatic illness. However, higher parasitological efficacy would imply higher clinical efficacy, as well as providing greater spillover benefit in terms of reducing transmission. Fifth, this analysis was restricted to a single SMC cycle, as multi-cycle individual-level data were not available for pooled analysis. However, efficacy is not expected to vary substantially across cycles because desethylamodiaquine will not accumulate over multiple cycles (see simulated profiles in Fig. S15 based on an existing PK model^23^).

This pooled-analysis of three studies of SPAQ as SMC in West and East Africa suggests that the parasitological efficacy of SPAQ is sub-optimal. Desethylamodiaquine concentrations are too low in around half of all children. Sulfadoxine-pyrimethamine appears to contribute little to the overall parasitological efficacy. Randomised studies of alternatives to amodiaquine and of the added benefit of sulfadoxine-pyrimethamine should be conducted in the Sahel and in other countries considering SMC deployment.

## Methods

### Data

All three studies were sponsored by the Malaria Consortium. The Mbale Regional Referral Hospital-REC (reference: MRRH-2022-168) and the Uganda National Council for Science and Technology (UNCST; reference: HS2212ES) approved the study in Uganda; the Comité Nacional de Bioética para Saúde, Mozambique Ministry of Health (reference 130/CNBS/22) and the Research Ethics Committee of the Hospital Clinic of Barcelona (reference 16/2021) approved the study in Mozambique; the ethics committee for health research in Burkina Faso (CERS) approved the study in Burkina Faso (reference N° 2022-07-167). In all three studies, the parents or carers gave fully informed written consent and declared that they were willing and able to comply with the study protocol and blood sampling. In Burkina Faso, due to lack of clarity in the initial information form regarding blood sample collection and processing, a second consent was re‑obtained after ethics committee approval to address incomplete information regarding the planned use of blood analysis results. The procedures were successfully implemented, and data from participants who could not be traced were excluded from the dataset prior to analysis.

This pooled analysis used individual-level data from these three observational studies evaluating seasonal malaria chemoprevention (SMC) with sulfadoxine–pyrimethamine plus amodiaquine conducted in Uganda (NCT05323721), Mozambique (NCT05186363), and Burkina Faso (NCT05478954). The full trial protocols are available with the underlying publications of the individual studies. The three sites included in this analysis represent contrasting resistance profiles: Uganda and Mozambique are East and Southern African settings where quintuple mutants predominate, while Burkina Faso is a West African setting where quadruple mutants are predominant, retaining greater susceptibility to sulfadoxine. The three study sites are all high malaria transmission settings^24^. Uganda experiences stable, perennial malaria transmission across 95% of the country^25^, while Mozambique bears one of the highest malaria burdens in Eastern and Southern Africa, with transmission occurring year-round^26^. Burkina Faso, by contrast, experiences intense but markedly seasonal transmission concentrated in the rainy season^27^.

The study designs and methods for the Uganda and Mozambique components have been previously published^8,9^, key methods are summarised below. The Mozambique enrolment count differs slightly from the previously published trial report due to use of an updated participant list. Briefly, across all sites, children aged 3–59 months were enroled at the time of SMC administration and followed through a standardised sampling schedule comprising blood sample collection at day 0 (pre-dose), day 7, and day 28. Each child contributed up to three blood samples, collected at distinct timepoints. Blood samples were used to quantify *Plasmodium* parasitaemia by qPCR (with LOD of 862 genome equivalent parasites/mL) and, where sufficient DNA was available, to perform molecular genotyping of drug resistance markers (*pfcrt*, *pfmdr1*, *pfdhfr*, *pfdhps*, and *pfaat1*, Table S3). Day 7 and day 28 samples were used to measure plasma concentrations of sulfadoxine, pyrimethamine, amodiaquine, and desethylamodiaquine, with day 28 samples additionally used to assess parasitaemia. Drug administration was fully observed in Uganda, but only the first dose of SP and AQ was directly observed in Burkina Faso and Mozambique.

### Analysis population

The analysis population comprised children who received SMC and had complete pharmacokinetic and parasitological data across the scheduled follow-up visits (days 7 and 28). To ensure appropriate interpretation of drug exposure, any samples suspected of having received another dose were excluded from all analyses. Such cases were identified based on either (i) amodiaquine concentrations exceeding 10 ng/mL on day 28, or (ii) higher drug levels on day 28 compared to day 7.

The primary efficacy analysis was restricted to children with blood samples available at all three timepoints (day 0, day 7, day 28), as the qPCR-confirmed malaria outcome was assessed at day 28 and required drug concentration data across the dosing interval for inclusion in the regression model. To provide a more complete picture of drug exposure across the study population, descriptive summaries of drug concentrations among all children with an available sample at each timepoint are presented in Table S2.

### Statistical analysis

#### Primary and secondary efficacy outcomes

The primary outcome was malaria infection by day 28, defined as detectable parasitaemia by PCR on day 28 or any episode of intercurrent clinical malaria between day 0 and day 28, defined as fever (axillary temperature ≥37.5 °C or history of fever within 24 hours) with a positive RDT. A sensitivity analysis fitted the same primary analysis model but treating all Ct values > 35 as negative. We used the same primary outcome as in the previous analysis of the Ugandan SMC study^8,9^.

Cycle threshold (Ct) values from qPCR at day 28 were analysed as a secondary outcome to explore the association between post-treatment drug exposure and parasite burden. Lower Ct values indicate higher parasite density. Ct values of 40 or above were treated as left-censored.

All statistical tests were two-sided with a significance threshold of *p* < 0.05. No adjustment for multiple comparisons was made as analyses were pre-specified.

#### Exposure

Drug exposure was evaluated using post-treatment concentrations of sulfadoxine, pyrimethamine, amodiaquine, and desethylamodiaquine measured on day 7 and day 28. For pyrimethamine, due to its short half-life, only day 7 concentrations were available (concentrations on day 28 were mostly below the LLOQ). For amodiaquine, concentrations were frequently quantifiable at day 7 but declined rapidly, with most values below the lower limit of quantification by day 28, aside from a small number of outliers. For sulfadoxine and desethylamodiaquine, additional pharmacokinetic metrics were derived for each child, including the area under the concentration–time curve (AUC) from day 7 to day 28 and the terminal elimination half-life. The AUC was calculated using the log-transformed concentrations at the two sampling times and the trapezoidal rule, representing the average exposure over the interval. Terminal half-life was estimated from the log-linear decline between the two time points. All exposure metrics were standardised (z-score) to allow comparison across drugs.

#### Primary exposure-efficacy analysis: malaria infection by day 28

To investigate the relationship between SMC drug exposure and the probability of malaria infection by day 28, we fitted generalized additive models (GAMs) with binomial link functions. Analyses were first conducted separately for desethylamodiaquine and sulfadoxine, followed by a combined model incorporating the selected exposure proxies for both drugs and day 7 pyrimethamine concentration.

For each drug, multiple pharmacokinetic metrics were evaluated as potential predictors of protection (Fig. S6) in a univariable GAM adjusted for study site, baseline malaria infection, nutritional status (z-MUAC), and smooth functions of age and enrolment time stratified by site. Akaike’s information criterion (AIC) was used to identify the most informative exposure proxy for each drug.

Based on this selection process, the standardized day 28 desethylamodiaquine concentration and the standardized day 7 pyrimethamine concentration were included together in a multivariable GAM to assess their combined influence on malaria risk. All models were fitted using restricted maximum likelihood (REML) with automatic smooth-term selection to penalise overfitting and shrink non-informative terms. To reduce undue influence from extreme values, analyses were restricted to observations with |z-score|<3 for day 28 desethylamodiaquine and |z-score | <4 for day 7 sulfadoxine.

To evaluate whether the protective effect of each drug varied with age, additional models were fitted separately for desethylamodiaquine, sulfadoxine, and pyrimethamine, each including an interaction term between the standardized drug concentration and age. These interaction models retained the same adjustment set as the primary analyses.

#### Secondary analysis: parasite density (Ct values)

For the secondary outcome, generalised additive models (GAMs) with Gaussian errors and an identity link were used to flexibly identify dominant drug effects and potential interactions, adjusting for age, site, baseline Ct, mid-upper arm circumference, and enrolment time. To appropriately account for left-censoring at Ct = 40, Gaussian Tobit regression models^28^ were fitted. Primary exposure variables included standardised (z-scaled) concentrations of desethylamodiaquine at day 28, sulfadoxine and pyrimethamine at day 7.

#### Sensitivity analysis

To estimate the causal effect of day 28 desethylamodiaquine exposure on malaria outcome, we applied inverse probability weighting based on the propensity score. Low versus high desethylamodiaquine exposure was defined using a threshold of 24 ng/ml as the population mean exposure. The propensity score, defined as the probability of low exposure conditional on baseline covariates, was estimated using logistic regression including age, nutritional status (MUAC z-score), sex, and study site. Stabilised inverse probability weights were constructed and applied to create a pseudo-population in which measured confounders were balanced across exposure groups. The primary causal effect was then estimated using a doubling robust weighted regression model including the same covariates in the outcome model.

### Reporting summary

Further information on research design is available in the Nature Portfolio Reporting Summary linked to this article.

## Supplementary information

Supplementary Information

Reporting Summary

Transparent Peer Review file

## Data Availability

Pseudonymised participant data used in this analysis are available for access via the WWARN website (https://www.iddo.org/wwarn/accessing-data). As users of a third-party dataset, the authors are not authorised to share the data independently. Requests for access will be reviewed by a data access committee to ensure that use of data protects the interests of the participants and researchers according to the terms of ethics approvals and the principles of equitable data sharing. Access is open to researchers for scientific research purposes, subject to signing a Data Use Agreement. Requests can be submitted by email to malariaDAC@iddo.org via the data access form. Decisions on access requests are typically communicated within 8 weeks of submission. Data access is granted for 1 year, renewable upon request.

## Code availability

All analyses were conducted in R (version 4.4.3). GAMs were fitted using the mgcv package (version 1.9-3)^29^ and left-censored Tobit models were fitted using the survival package (version 3.8-3)^30^. Propensity score–based inverse probability weighting was used in sensitivity analyses, implemented using the ipw (version 1.2.2) and survey packages (version 4.4-2)^31^. All analysis code used to generate the results and figures in this manuscript is available on GitHub (https://github.com/Chayaninss/SMC-Pooled-analysis) and has been deposited on Zenodo with https://doi.org/10.5281/zenodo.22114310^32^.
