# Supplementary material for: Parasitological efficacy of sulfadoxine-pyrimethamine and amodiaquine for seasonal malaria chemoprevention in Africa
Source: Nat Commun. 2026 Oct 1;17:11263. doi: 10.1038/s41467-026-78189-2 (PMC13720088; doi:10.1038/s41467-026-78189-2)
Supplement: Supplementary file 1 [file 41467_2026_78189_MOESM1_ESM.pdf]

Parasitological efficacy of sulfadoxine-pyrimethamine and amodiaquine for Seasonal Malaria Chemoprevention in Africa

Supplementary Information

Chayanin Sararat[1,2,*], Paul Sondo[3,4], Craig Bonnington[2], Jane Achan[5], Chukwudi A Nnaji[5], Anna Y Burkhard[5], Gauthier Tougri[6], Adama Traore[7], Aimé Parfait Korou Yaro[7], Anthony Nuwa[8], Richard Kajubi[8], Kanokon Suwannasin[1], Stephane Proux[9], Urairat Koesukwiwat[1], Sonia Enosse[10], Ivan Alejandro Pulido Tarquino[10], Stije J Leopold[1,2], Arjen M Dondorp[1,2], Halidou Tinto[4], James K Tibenderana[5], Joel Tarning[1,2,11], Mallika Imwong[1,2,12], Francois H Nosten[2,9], James A Watson[2,3,#], Nicholas J White[1,2,#,†]

1: Mahidol-Oxford Tropical Medicine Research Unit, Faculty of Tropical Medicine, Mahidol University, Bangkok 10400, Thailand; 2: Centre for Tropical Medicine and Global Health, Nuffield Department of Medicine, University of Oxford, OX3 7LJ, UK; 3: Infectious Diseases Data Observatory, Oxford, UK; 4: Institut de Recherche en Sciences de la Santé- Clinical Research Unit of Nanoro (CRUN), Burkina Faso; 5: Malaria Consortium, London, UK; 6: Programme National de Lutte contre le Paludisme, Ouagadougou, Burkina Faso; 7: Malaria Consortium, Ouagadougou, Burkina Faso; 8: Malaria Consortium, Kampala, Uganda; 9: Shoklo Malaria Research Unit (SMRU), Mahidol Oxford Tropical Medicine Research Unit, Faculty of Tropical Medicine, Mahidol University, Mae Sot, Thailand; 10: Malaria Consortium, Maputo, Mozambique; 11: Division of Clinical Pharmacology, Department of Laboratory Medicine, Karolinska Institutet, Stockholm, Sweden; 12: Department of Molecular Tropical Medicine and Genetics, Faculty of Tropical Medicine, Mahidol University, Bangkok, Thailand.

[*]For correspondence: chayanin@tropmedres.ac; †deceased; [#]These authors jointly supervised this work

Supplementary Results

Site-specific association between SP component concentrations and day 28 Ct

To explore the influence of pfdhps K540E resistance on the independent contributions of SP components, site-specific associations between SP component concentrations and day 28 Ct were derived from a model including sulfadoxine×site and pyrimethamine×site interaction terms. For sulfadoxine, a significant positive association with day 28 Ct was observed only in Burkina Faso (β = 0.83, 95% CI 0.09 to 1.57, p = 0.027), where pfdhps K540E is largely absent, while no significant sulfadoxine-Ct association was detected in Uganda (β = 0.22, 95% CI −0.57 to 1.00, p = 0.589) or Mozambique (β = 0.78, 95% CI −0.56 to 2.12, p = 0.253). For pyrimethamine, no significant site-specific associations were detected at any site (Uganda: β = 0.14, 95% CI −0.73 to 1.01, p = 0.752; Mozambique: β = −0.75, 95% CI −2.10 to 0.59, p = 0.272; Burkina Faso: β = 0.45, 95% CI −0.38 to 1.27, p = 0.285).

Site-specific concentration-dependent attenuation of desethylamodiaquine efficacy

To evaluate whether this attenuation differed across sites, a proxy for pfdhps K540E resistance status, we tested for site-by-SP concentration interactions in a left-censored Tobit model. No significant overall difference was detected across sites (likelihood ratio test: deviance = 6.20, df = 6, p = 0.401). However, site-specific estimates derived from a three-way desethylamodiaquine×sulfadoxine×site interaction model demonstrated significant attenuation in Uganda (β = −0.85, 95% CI −1.61 to −0.10, p = 0.027) and Burkina Faso (β = −0.90, 95% CI −1.46 to −0.35, p = 0.001) with consistent but non-significant estimate in Mozambique (β = −0.61, 95% CI −2.10 to 0.87, p = 0.419), likely reflecting limited statistical power at that site (n = 104).

Sensitivity analysis: alternative Ct threshold for PCR positivity

To access the robustness of the primary analysis to the choices of Ct threshold for defining PCR positivity, we repeated the multivariable logistic regression using stricter threshold of Ct < 35 as the definition of a positive

result, thereby excluding low-density infections that may represent residual non-viable parasite DNA. The estimated exposure-response relationships were consistent with the primary analysis for all three drug components: desethylamodiaquine OR = 0.46 (95% CI 0.36–0.58), sulfadoxine OR = 0.84 (95% CI 0.65–1.07), and pyrimethamine OR = 0.86 (95% CI 0.65–1.13), compared with OR = 0.55 (95% CI 0.44–0.68), OR = 0.80 (95% CI 0.63–1.00), and OR = 0.88 (95% CI 0.67–1.20) in the primary analysis, respectively.

Supplementary Tables

Table S1: Summary of malaria infection in the analysis population versus all available samples. Percentage of malaria infection, any species, count over samples available for PCR, while species-specific infection percentage counted over PCR-malaria positive subjects

Species	Site	Baseline	Day-28 follow-up
**Efficacy analysis population**			
All	Overall	478/934 (51.2%)	284/934 (30.4%)
All	Uganda	239/407 (58.7%)	109/407 (26.8%)
All	Mozambique	68/104 (65.4%)	44/104 (42.3%)
All	Burkina Faso	171/423 (40.4%)	131/423 (31.0%)
Pf	Overall	435/478 (91%)	238/284 (83.8%)
Pf	Uganda	223/239 (93.3%)	85/109 (78%)
Pf	Mozambique	63/68 (92.6%)	41/44 (93.2%)
Pf	Burkina Faso	149/171 (87.1%)	112/131 (85.5%)
Po	Overall	86/478 (18%)	29/284 (10.2%)
Po	Uganda	47/239 (19.7%)	22/109 (20.2%)
Po	Mozambique	25/68 (36.8%)	1/44 (2.3%)
Po	Burkina Faso	14/171 (8.2%)	6/131 (4.6%)
Pm	Overall	11/478 (2.3%)	1/284 (0.4%)
Pm	Uganda	10/239 (4.2%)	1/109 (0.9%)
Pm	Mozambique	0/68 (0%)	0/44 (0%)
Pm	Burkina Faso	1/171 (0.6%)	0/131 (0%)
Pv	Overall	0/478 (0%)	0/284 (0%)
Pv	Uganda	0/239 (0%)	0/109 (0%)
Pv	Mozambique	0/68 (0%)	0/44 (0%)
Pv	Burkina Faso	0/478 (0%)	0/284 (0%)

Full available population

All	Overall	634/1191 (53.2%)	316/1020 (31%)
All	Uganda	245/426 (57.5%)	110/414 (26.6%)
All	Mozambique	213/327 (65.1%)	75/178 (42.1%)
All	Burkina Faso	176/438 (40.2%)	131/428 (30.6%)
Pf	Overall	580/634 (91.5%)	262/316 (82.9%)
Pf	Uganda	228/245 (93.1%)	85/110 (77.3%)
Pf	Mozambique	198/213 (93%)	65/75 (86.7%)
Pf	Burkina Faso	154/176 (87.5%)	112/131 (85.5%)
Po	Overall	133/634 (21%)	30/316 (9.5%)
Po	Uganda	48/245 (19.6%)	22/110 (20%)
Po	Mozambique	71/213 (33.3%)	2/75 (2.7%)
Po	Burkina Faso	14/176 (8%)	6/131 (4.6%)
Pm	Overall	12/634 (1.9%)	1/316 (0.3%)
Pm	Uganda	10/245 (4.1%)	1/110 (0.9%)
Pm	Mozambique	1/213 (0.5%)	0/75 (0%)
Pm	Burkina Faso	1/176 (0.6%)	0/131 (0%)
Pv	Overall	2/634 (0.3%)	0/316 (0%)
Pv	Uganda	0/245 (0%)	0/110 (0%)
Pv	Mozambique	2/213 (0.9%)	0/75 (0%)
Pv	Burkina Faso	0/176 (0%)	0/131 (0%)

Table S2: Drug concentration summary (median, IQR) across the efficacy analysis population and full available population.

	Concentration (ng/mL)		
	**Uganda**	**Mozambique**	**Burkina Faso**
**Efficacy analysis population**			
**Sulfadoxine (Day 7)**	28 (22.2 - 38.7) $\times 10^3$	24.5 (16.1 – 32.7) $\times 10^3$	31.3 (21.0 - 41.5)$\times 10^3$
**Sulfadoxine (Day 28)**	1.6 <LLOQ - 3.7) $\times 10^3$	<LLOQ (<LLOQ - 1.7)$\times 10^3$	1.0 (<LLOQ - 3.2)$\times 10^3$
**Pyrimethamine (Day 7)**	91 (70 - 120)	46 (35 - 70)	49 (34 - 69)
**Pyrimethamine (Day 28)**	<LLOQ (<LLOQ - 6)	<LLOQ	<LLOQ

**Amodiaquine (Day 7)**	<LLOQ (<LLOQ - 5)	<LLOQ	3 (<LLOQ - 5)
**Amodiaquine (Day 28)**	<LLOQ	<LLOQ	<LLOQ
**Desethylamodiaquine (Day 7)**	155 (117 - 205)	99 (69 - 148)	98 (69 - 140)
**Desethylamodiaquine (Day 28)**	30 (18 - 46)	14 (9 - 24)	26 (17- 39)
**Full available population**			
**Sulfadoxine (Day 7)**	29 (22.3 - 38.5) $\times 10^3$	20.2 (13.7 – 30.5) $\times 10^3$	31.4 (21.0 - 41.4)$\times 10^3$
**Sulfadoxine (Day 28)**	1.6 <LLOQ - 3.7) $\times 10^3$	<LLOQ (<LLOQ - 1.2)$\times 10^3$	1.1 (<LLOQ - 3.2)$\times 10^3$
**Pyrimethamine (Day 7)**	92 (70 - 120)	44 (27 - 61)	49 (34 - 68)
**Pyrimethamine (Day 28)**	<LLOQ (<LLOQ - 6)	<LLOQ	<LLOQ
**Amodiaquine (Day 7)**	<LLOQ (<LLOQ - 5)	<LLOQ	3 (<LLOQ - 5)
**Amodiaquine (Day 28)**	<LLOQ	<LLOQ	<LLOQ
**Desethylamodiaquine (Day 7)**	156 (117 - 206)	88 (48 - 130)	98 (68 - 138)
**Desethylamodiaquine (Day 28)**	30 (18 - 46)	14 (9 - 23)	26 (17- 39)

LLOQ: lower limit of quantification (840 ng/mL for sulfadoxine, 4.42 ng/mL for pyrimethamine, 1.87 ng/mL for amodiaquine, and 2.95 ng/mL for desethylamodiaquine)

Table S3: Distribution of molecular resistance markers at baseline.

Gene	Position	Genotype	Frequencies of genotype status		
			**Uganda**	**Mozambique**	**Burkina Faso**
**pfdhps**	A437	A	0(0%)	1(2%)	3(4%)
		A/G	1(3%)	9(20%)	7(8%)
		G	33(97%)	36(78%)	74(88%)
	K540	K	0 (0%)	1(2%)	83(99%)
		E/K	0 (0%)	10(22%)	0 (0%)
		E	34(100%)	35(76%)	1(1%)
**pfdhfr**	N51	N	0 (0%)	0 (0%)	9(11%)
		I/N	0 (0%)	0 (0%)	14(17%)
		I	34(100%)	46(100%)	61(73%)
	C59	C	1(3%)	2(4%)	7(8%)
		C/R	8(24%)	3(7%)	28(33%)

		R	24(75%)	41(89%)	49(58%)
	S108	S	0 (0%)	0 (0%)	4(5%)
		N/S	0 (0%)	0 (0%)	10(12%)
		N	34(100%)	46(100%)	70(83%)
**Pfcrt**	C72	C	117(100%)	46(100%)	88(100%)
	V73	V	117 (100%)	46(100%)	88(100%)
	M74	M	117 (100%)	46(100%)	83(94%)
		I/M	0 (0%)	0 (0%)	4(5%)
		I	0 (0%)	0 (0%)	1(1%)
	N75	N	117 (100%)	46(100%)	83(94%)
		D/E	0 (0%)	0 (0%)	3(3%)
		E/N	0 (0%)	0 (0%)	1(1%)
		D/N	0 (0%)	0 (0%)	0 (0%)
		E	0 (0%)	0 (0%)	1(1%)
	K76	K	117 (100%)	46(100%)	83(94%)
		K/T	0 (0%)	0 (0%)	4(5%)
		T	0 (0%)	0 (0%)	1(1%)
**Pfmdr1**	N86	N	29(100%)	46(100%)	75(96%)
		N/Y	0 (0%)	0 (0%)	1(1%)
		Y	0 (0%)	0 (0%)	2(3%)
	Y184	Y	9(31%)	21(46%)	28(36%)
		F/Y	10(34%)	15(33%)	15(19%)
		F	10(34%)	10(22%)	34(44%)
	D1246	D	38(95%)	46(100%)	77(99%)
		D/Y	1(3%)	0 (0%)	1(1%)
**Pfaat1**	S258	S	42(52%)	0 (0%)	1(3%)
		S/L	20(25%)	0 (0%)	3(9%)
		L	19(23%)	0 (0%)	29(88%)
	F313	F	81(100%)	0 (0%)	33(100%)

Supplementary Figures

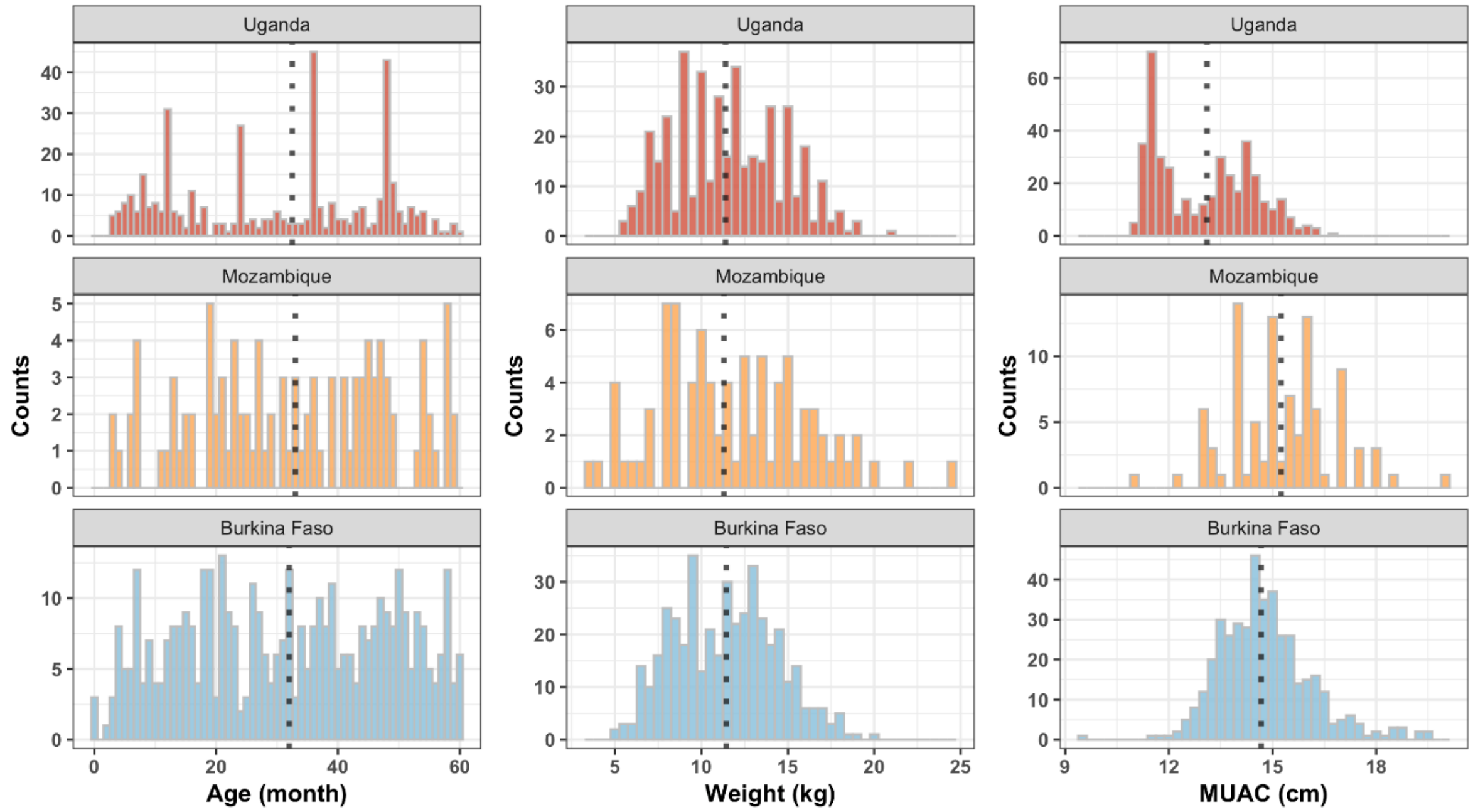

Figure S1: Distributions of demographic characteristics of the analysis population (n = 934). Dashed lines represent median values in each subpopulation.

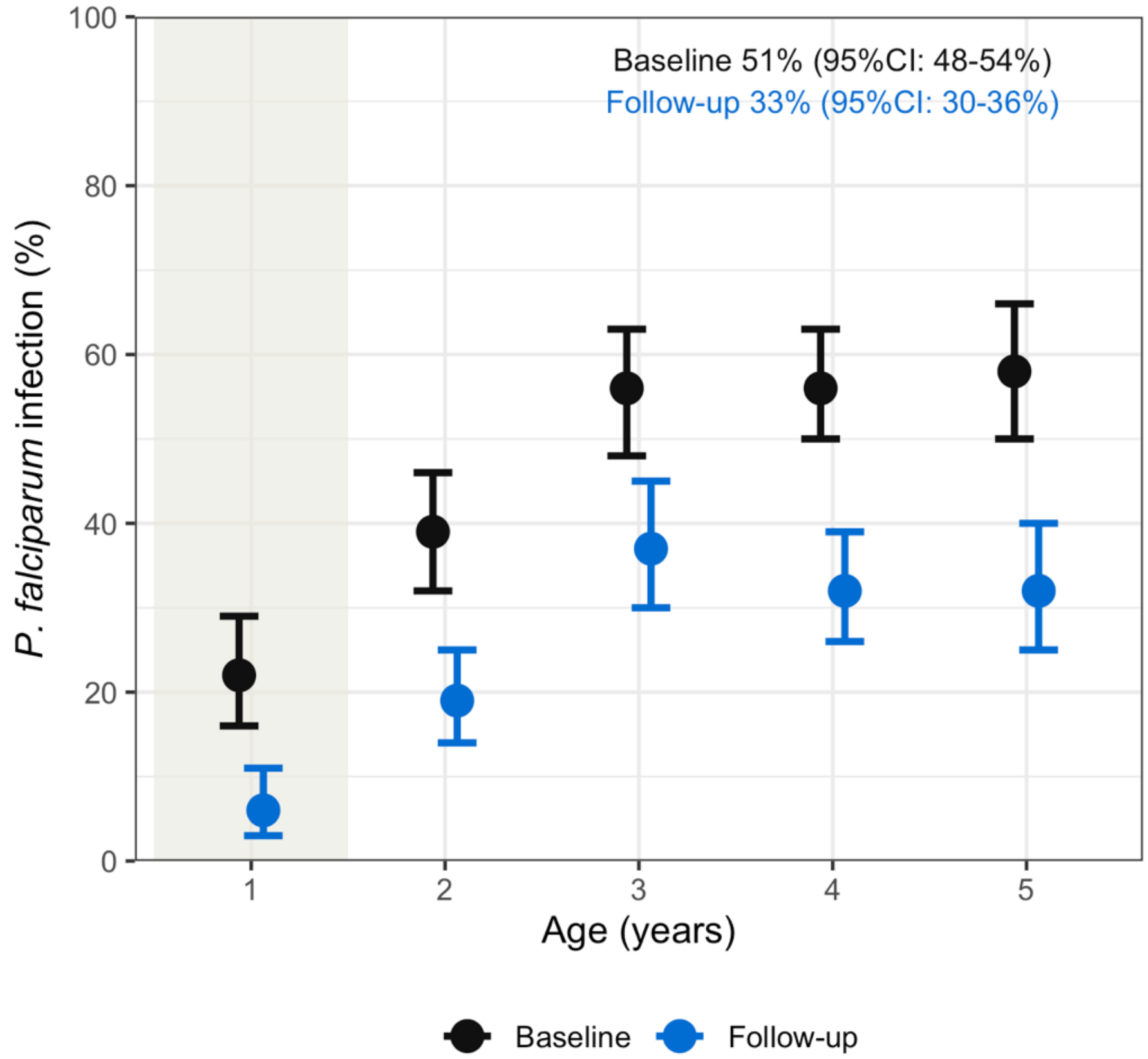

Figure S2: *Plasmodium falciparum*-specific infection prevalence by age (n = 934). Children aged 3–11 months received a half-dose of SPAQ. Prevalence point estimates are shown with 95% binomial confidence intervals.

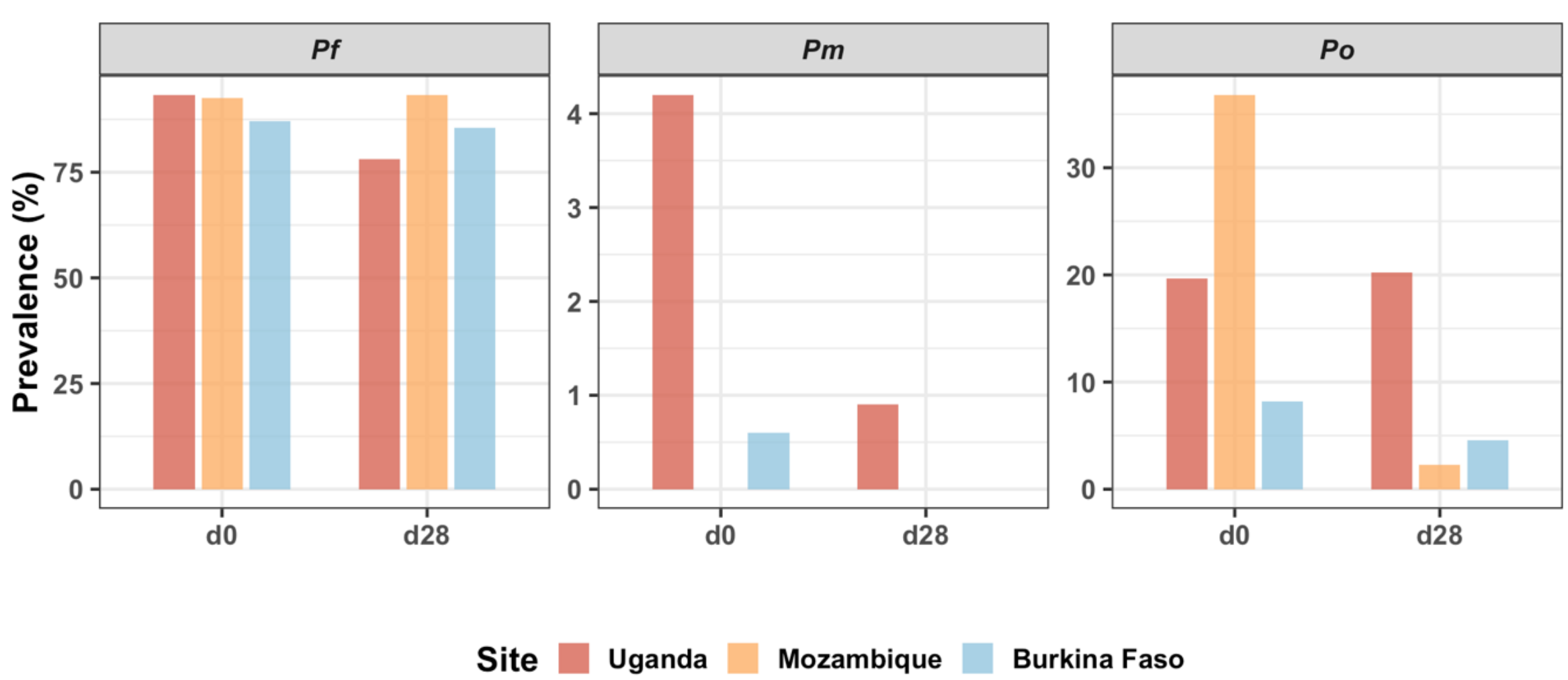

Figure S3: Plasmodium species prevalence (n = 934). Note that percentage may sum to more than 100% due to the presence of mixed-species infections.

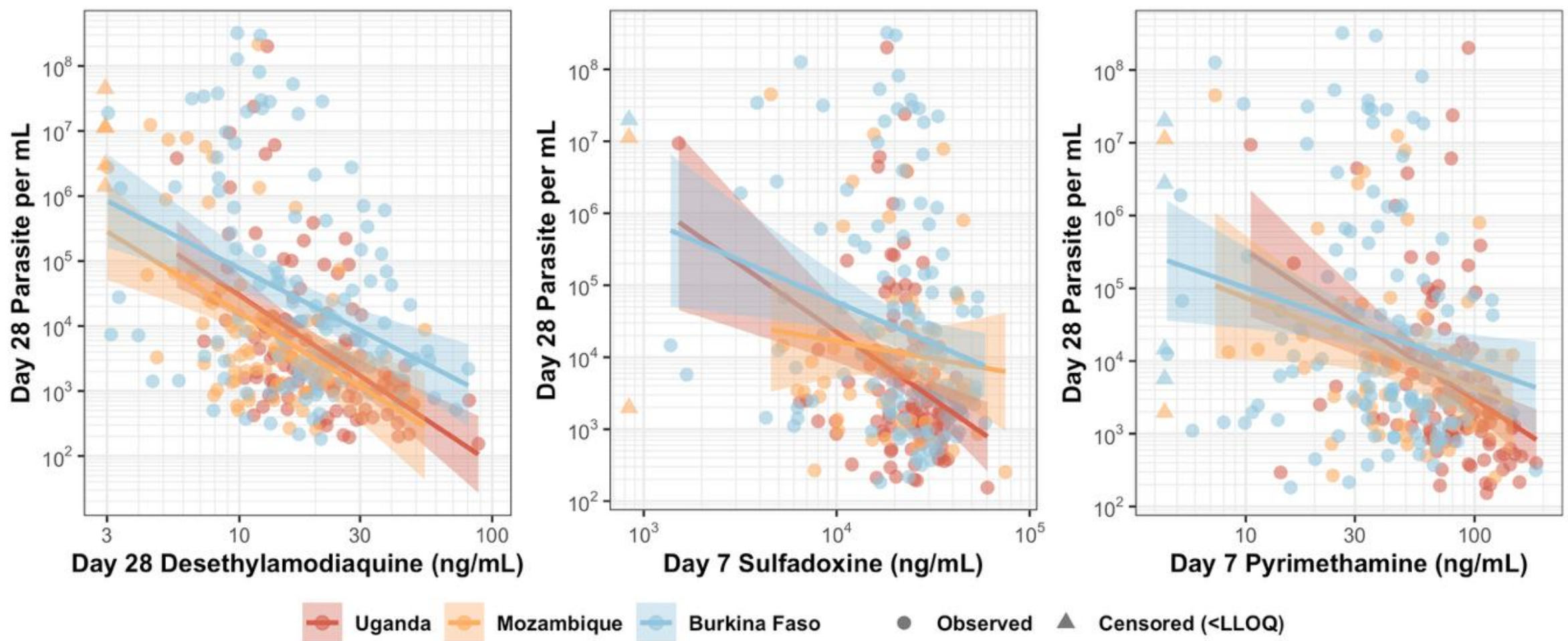

Figure S4: Relationship between qPCR parasite densities and post-treatment SMC drug concentrations (n = 934).

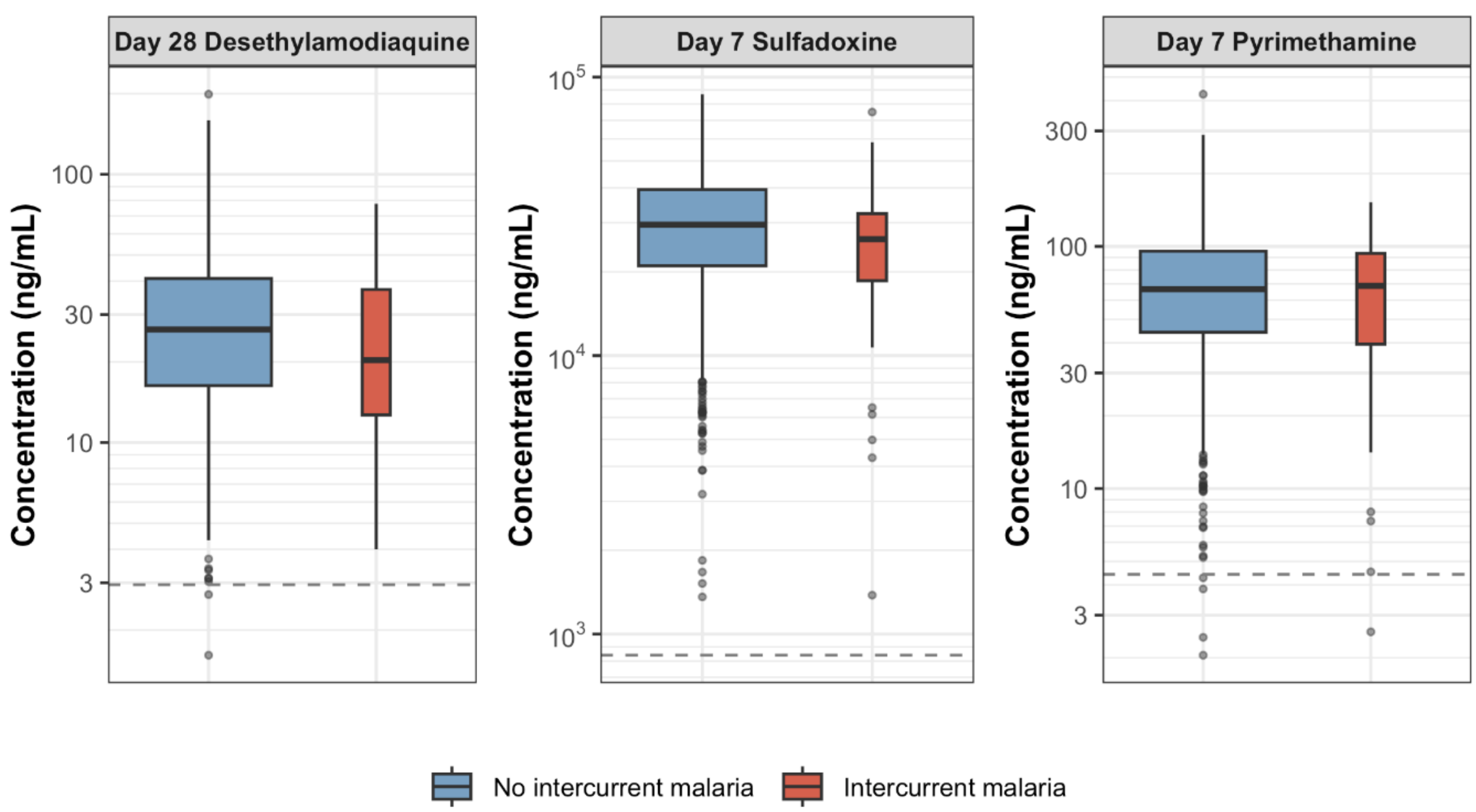

Figure S5. Antimalarial drug concentrations among children with and without intercurrent clinical malaria (n = 934). Boxplots of day 7 sulfadoxine, day 7 pyrimethamine, and day 28 desethylamodiaquine, comparing children who experienced intercurrent clinical malaria versus those who did not.

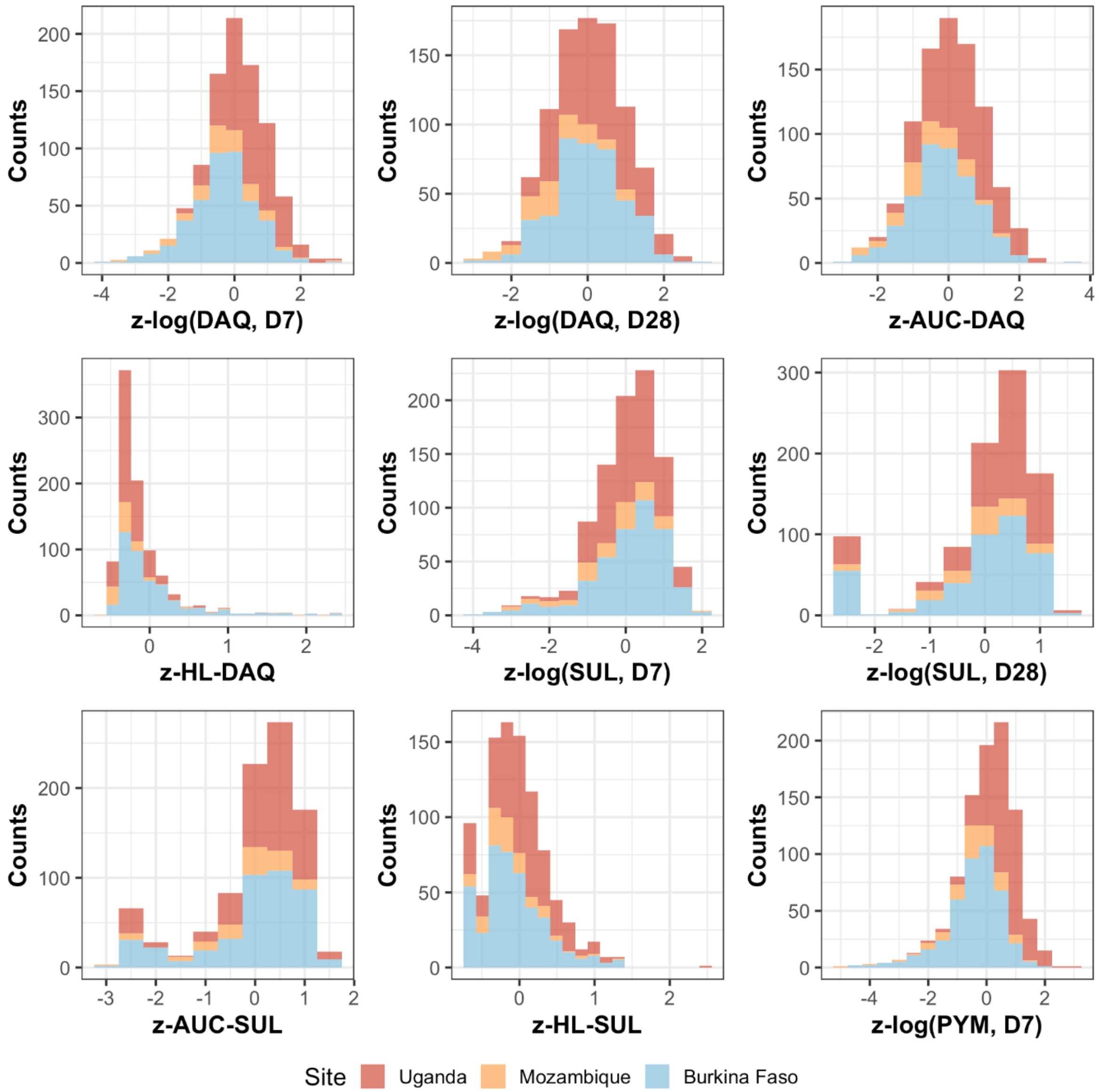

Figure S6: Distribution of standardized (z-score) exposure metrics for Desethylamodiaquine (DAQ), Sulfadoxine (SUL) and pyrimethamine (PYM) (n = 934). Metrics include standardized day 7 and day 28 concentrations, area under the concentration–time curve (AUC), and terminal half-life (HL), shown across the study population.

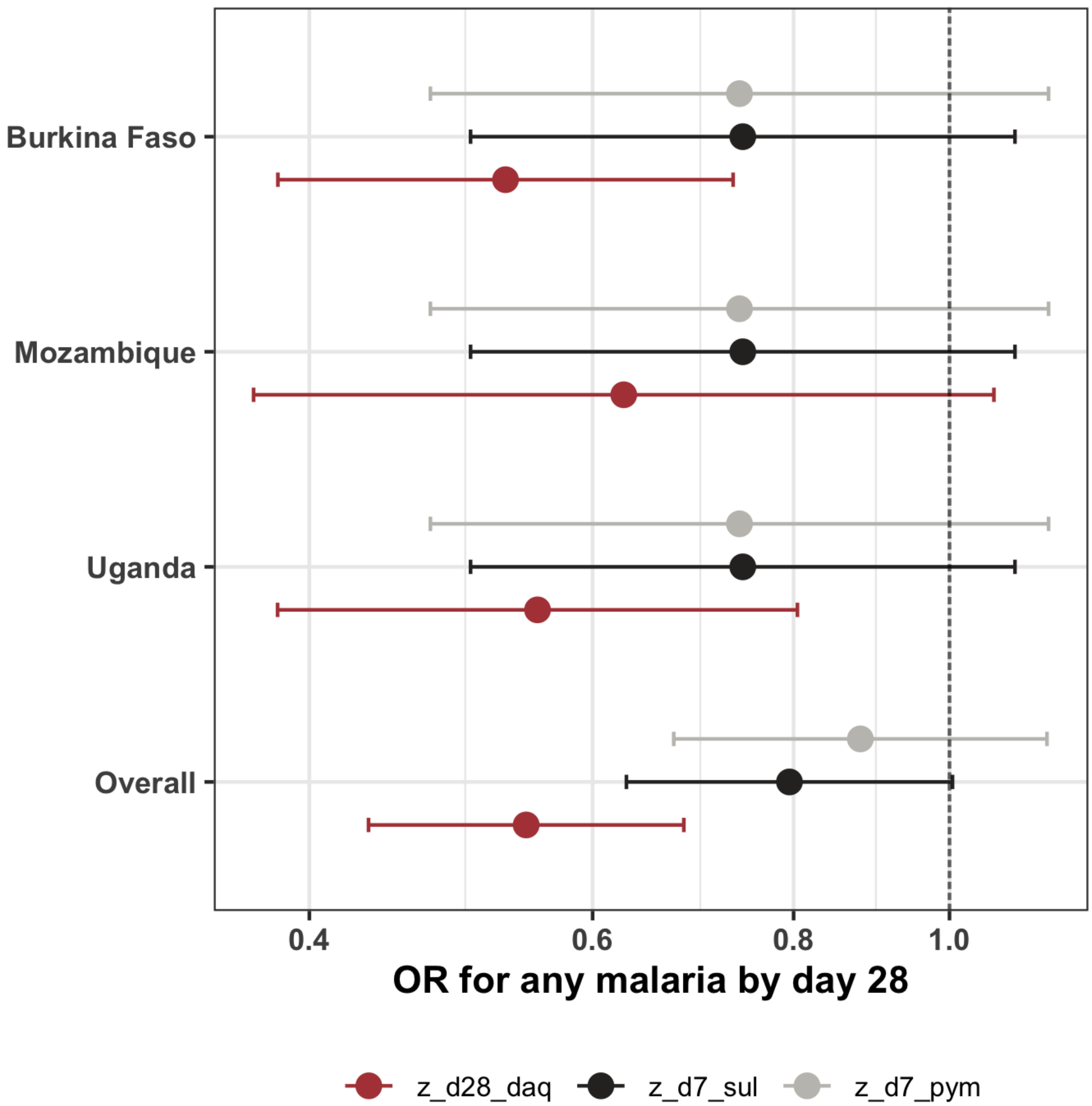

Figure S7: Odds ratios for malaria infection by day 28 in relation to post-treatment SMC drug exposure. Drug concentrations were log-transformed and standardised to allow comparison across drugs (n = 934). Drug concentrations were log-transformed and standardised to allow comparison across drugs. Odds ratios and 95% confidence intervals were estimated from univariable generalized additive models (GAMs) adjusted for study site, baseline malaria infection, nutritional status (z-MUAC), and smooth functions of age and enrolment time stratified by site, fitted using restricted maximum likelihood (REML).

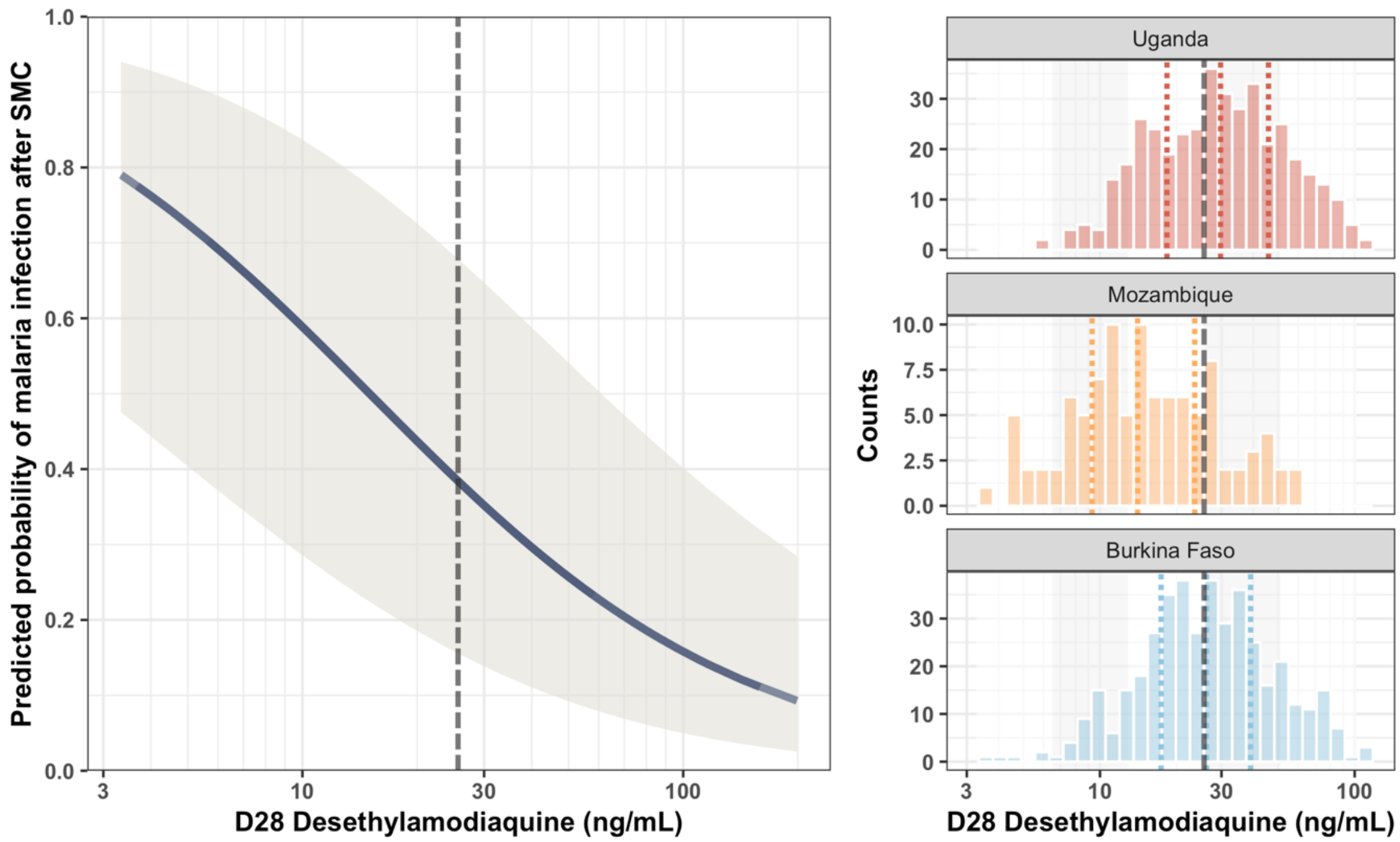

Figure S8: Predicted probability of malaria infection and post-treatment drug concentration distributions (n = 934). Predicted probability of malaria infection by day 28 is shown against concentrations of D28 Desethylamodiaquine calculated for a child of median age, weight, and MUAC who was malaria-negative at baseline. Histograms show concentration distributions by site. The black dashed line marks the overall mean, and coloured dashed lines denote site-specific medians and interquartile ranges. Shaded vertical stripes indicate one-standard-deviation intervals from the mean

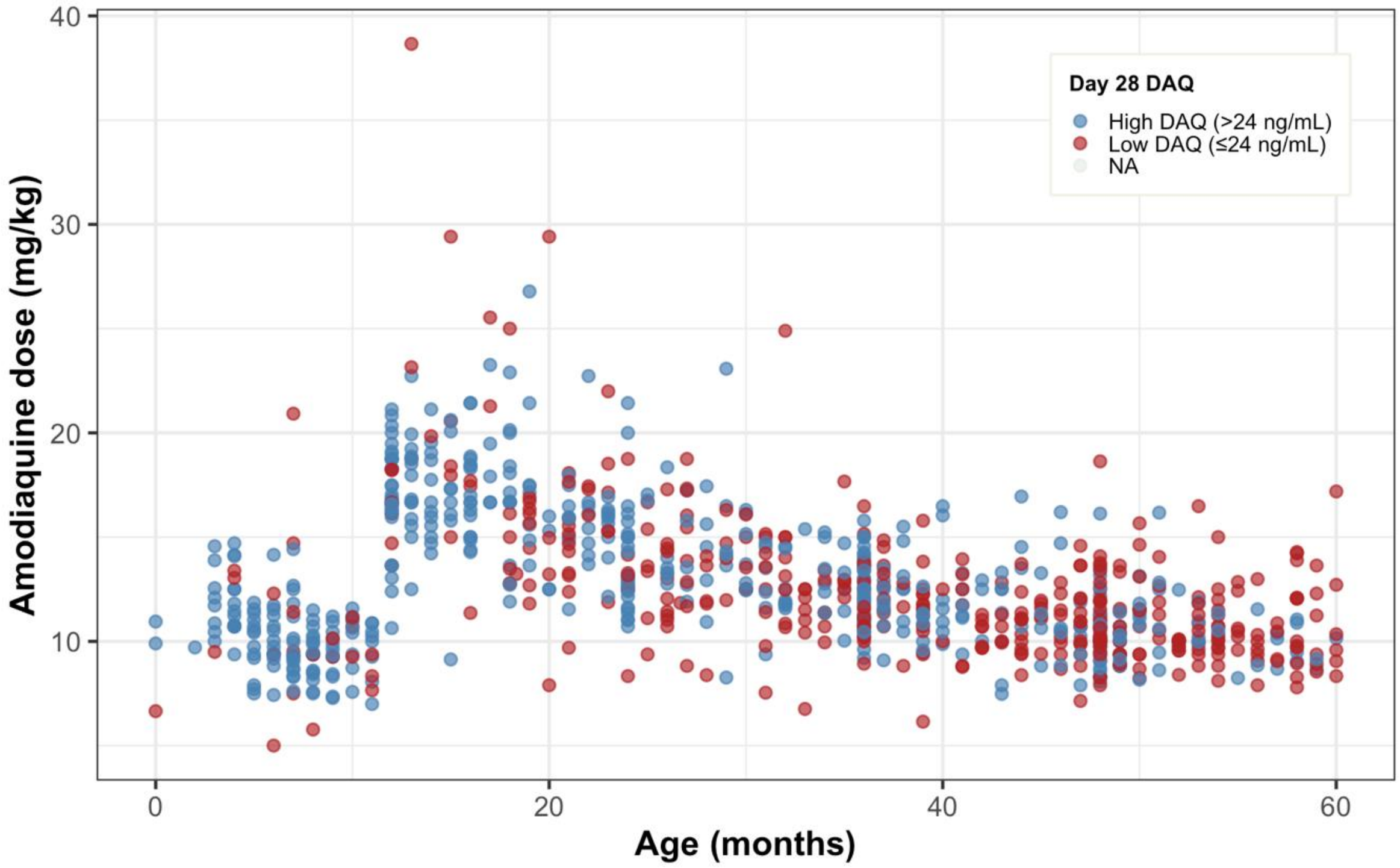

Figure S9: Amodiaquine dose per kilograms administered by age (n = 934). Children below one-year-old got 75 mg of amodiaquine (half dose) otherwise got 150 mg (full dose). Colours indicate whether day 28 desethylamodiaquine concentration was above or below the predefined threshold.

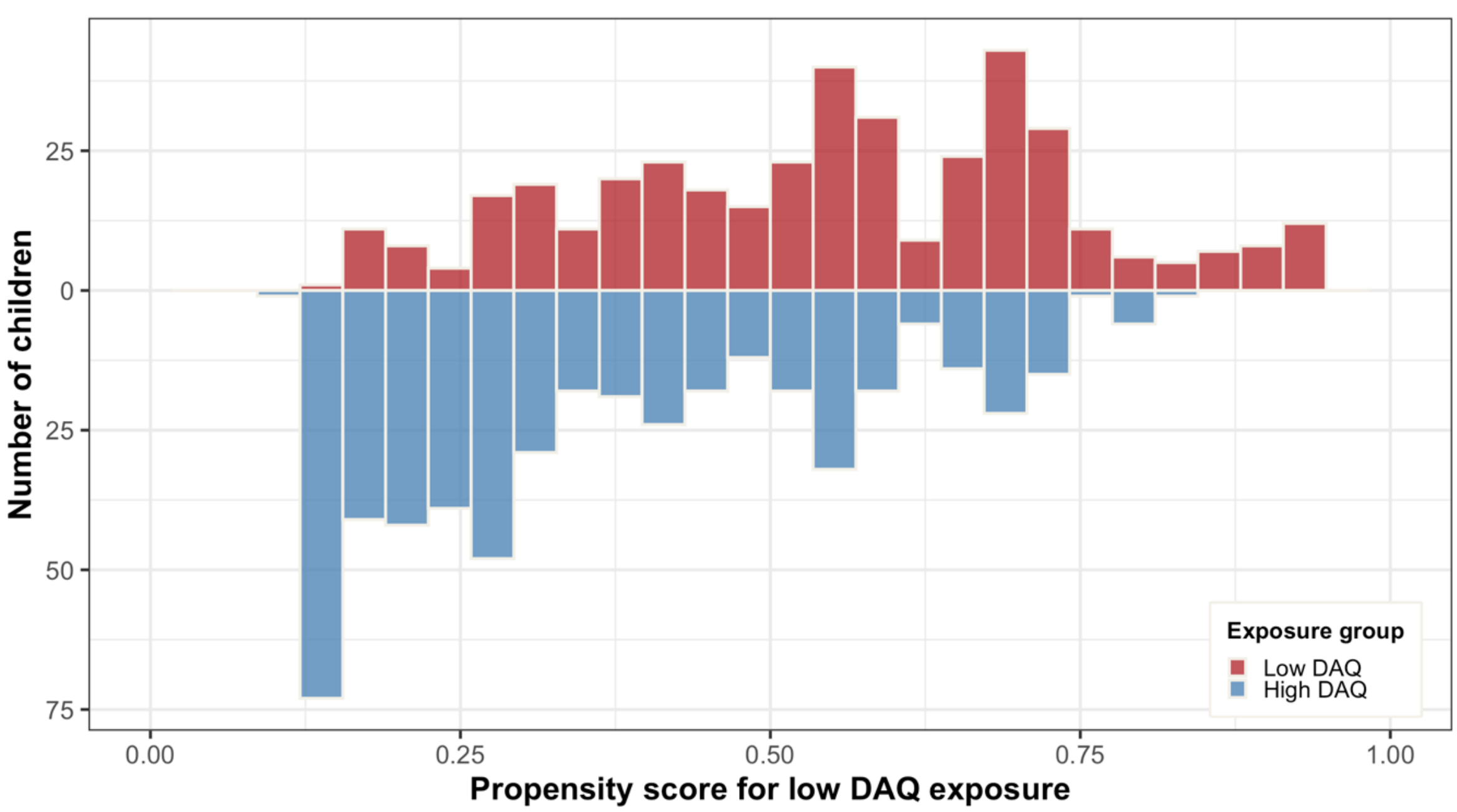

Figure S10: Propensity scores for low versus high day 28 desethylamodiaquine exposure (n = 934). Low exposure was defined as day 28 desethylamodiaquine concentration <24 ng/mL. Propensity scores represent the estimated probability of low exposure conditional on baseline covariates (age, MUAC z-score, sex, and study site), estimated using logistic regression.

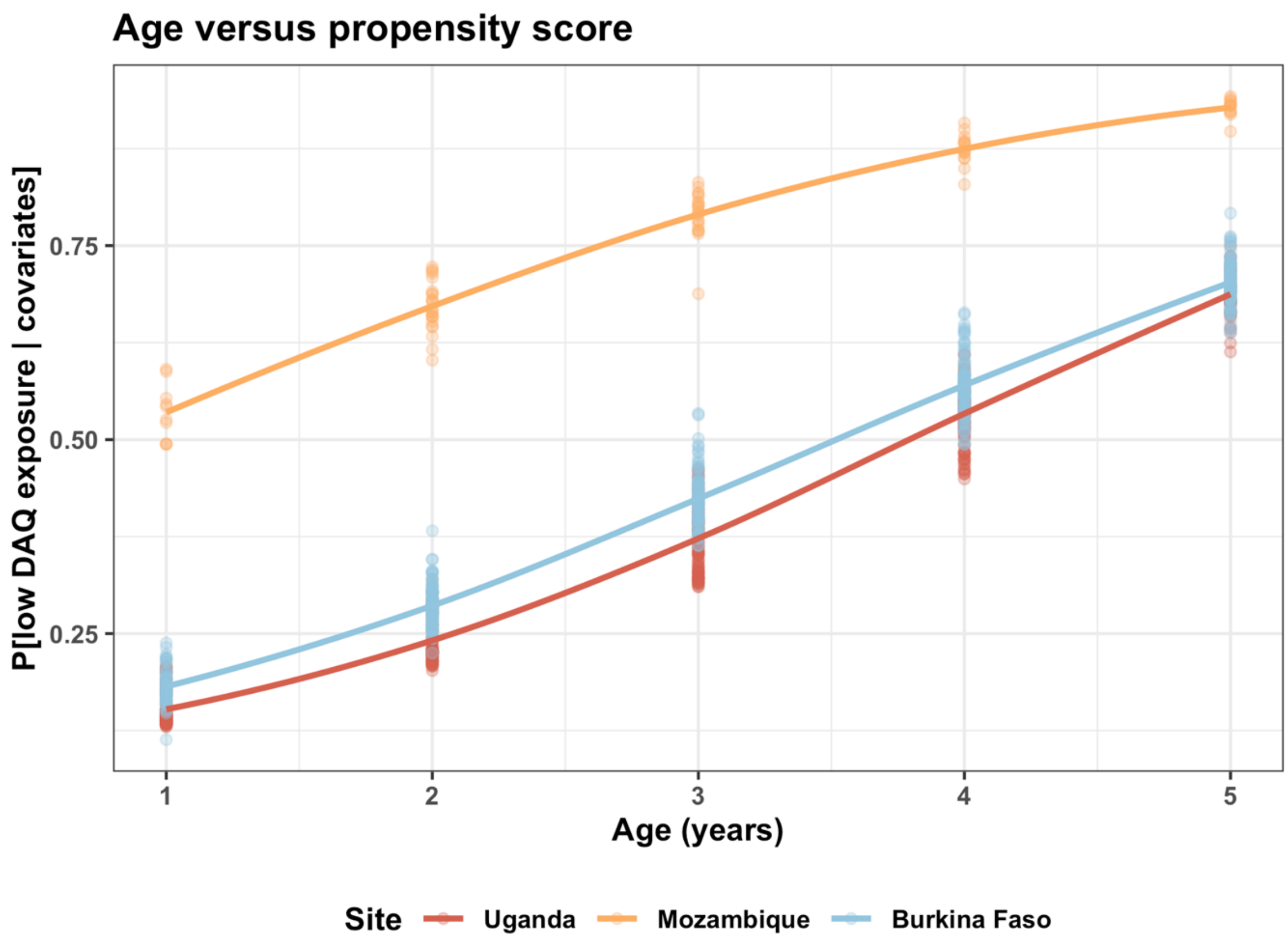

Figure S11. Propensity score for low day 28 desethylamodiaquine exposure (<24 ng/mL) by age (n = 934). Propensity scores were estimated using logistic regression including age, nutritional status (MUAC z-score), sex, and study site as covariates.

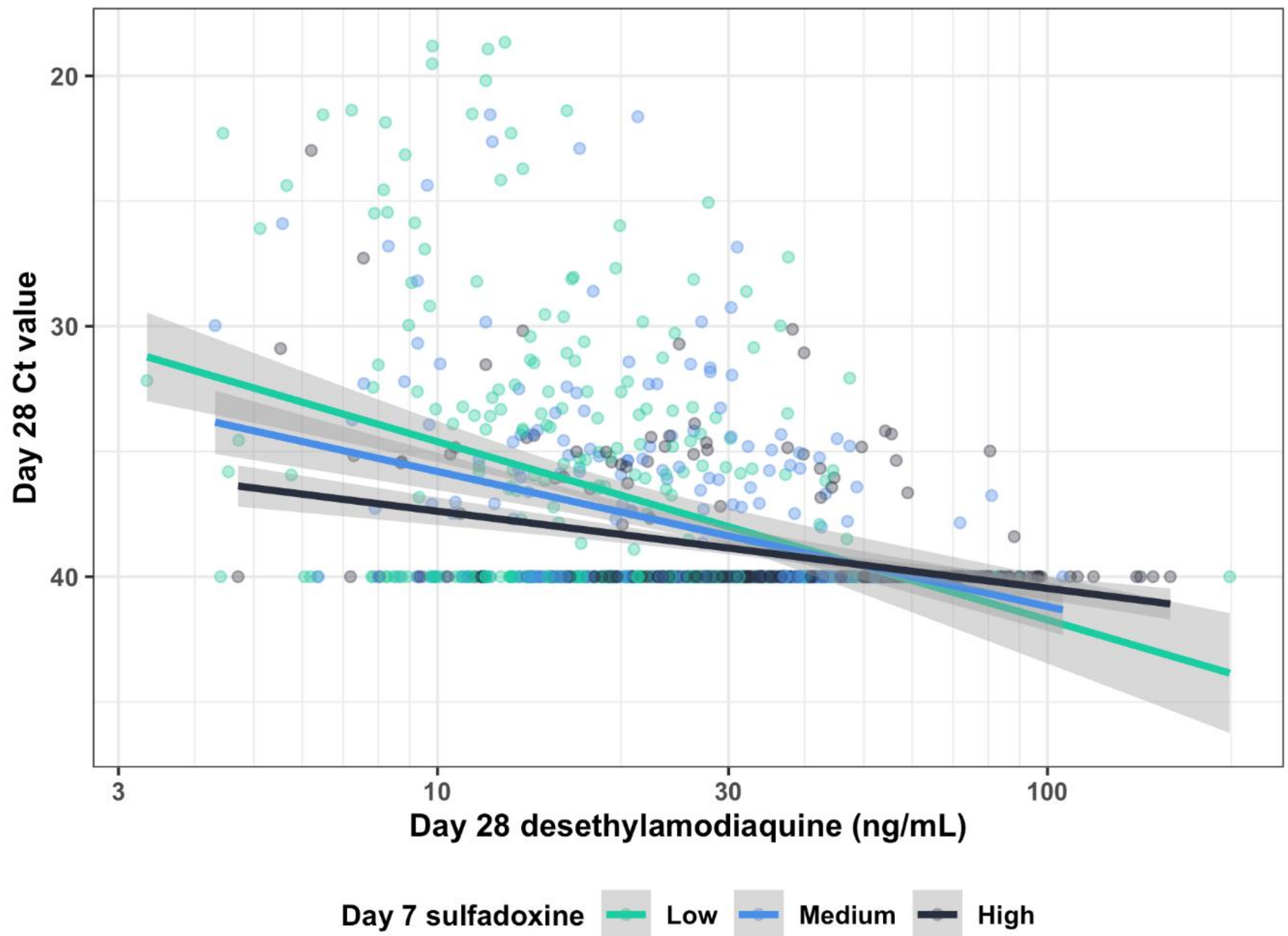

Figure S12: Concentration-dependent attenuation of desethylamodiaquine protective effect by sulfadoxine (n=934; analyses restricted to observations with |z-score| <3 for day 28 desethylamodiaquine). Scatter plot of day 28 Ct values against day 28 desethylamodiaquine concentrations, stratified by day 7 sulfadoxine concentration tertiles (low, medium, and high). The y-axis is reversed such that higher Ct values reflect lower parasitaemia. Lines represent linear regression fits with 95% confidence intervals for each sulfadoxine tertile. A steeper slope in the low sulfadoxine group indicates a stronger desethylamodiaquine protective effect at lower sulfadoxine concentrations.

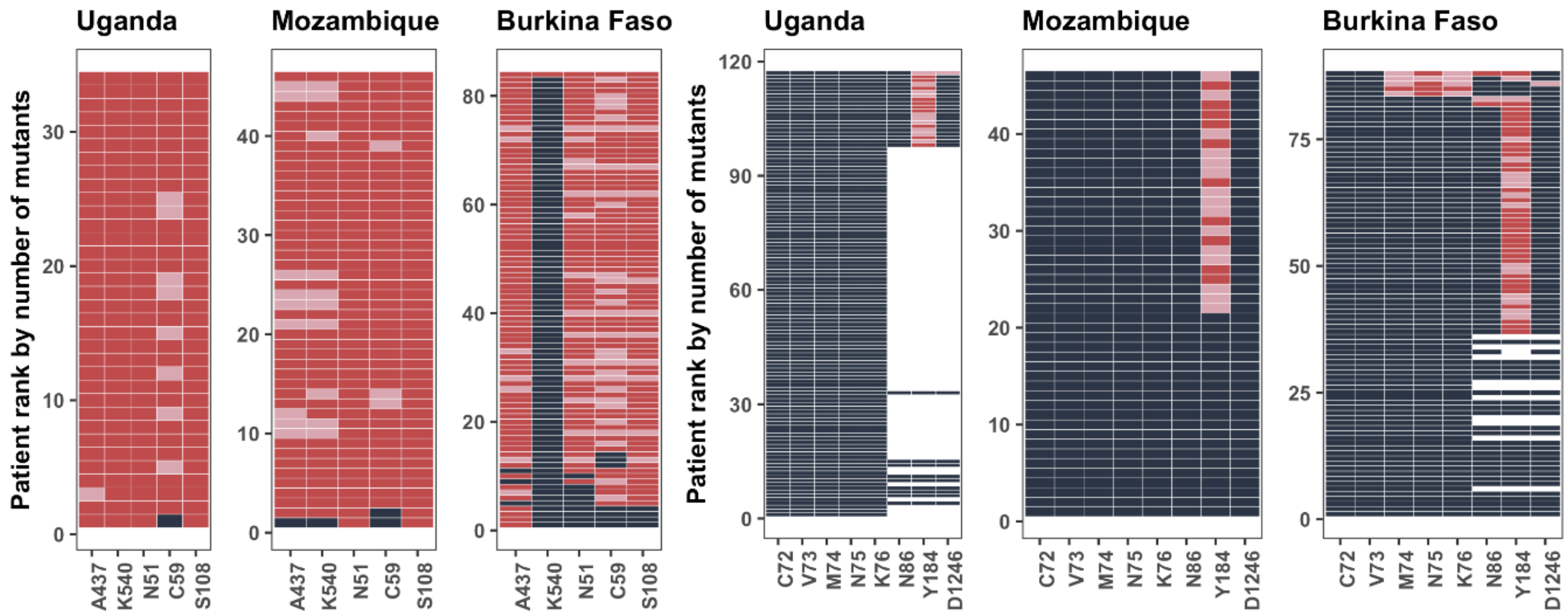

Figure S13: Individual-level genotyping of key SPAQ-resistance–associated mutations at baseline across study sites, ranked by the number of mutant codons (n = 251). Colours indicate genotype status (dark blue = pure wild-type, pink = mixed mutant, and red = pure mutant). Markers include positions in *pfdhps*, *pfdhfr*, *pfcrt*, and *pfmdr1*.

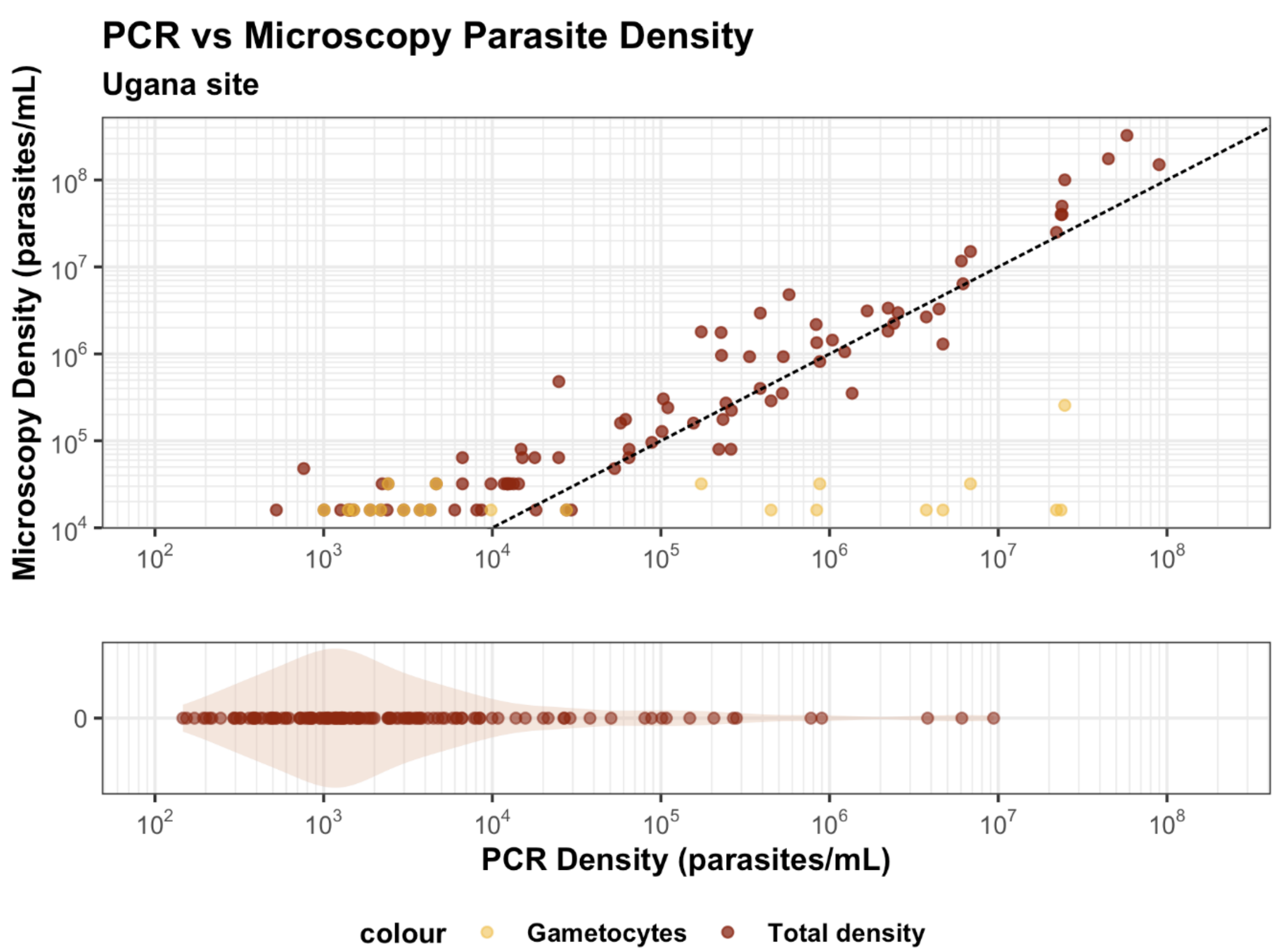

Figure S14: Agreement between PCR-estimated and microscopy-determined parasite densities at day-28 (restricted to Uganda site, n = 407). Blood slides were read by microscopy only for samples that were PCR positive, therefore no qPCR-negative/microscopy-positive samples are present. Total parasite density (red) and gametocyte density (yellow) as estimated by microscopy are shown separately. This comparison is

restricted to the Uganda site due to poor microscopy quality at the other two sites. The bottom panel shows the datapoints which were PCR positive but microscopy negative.

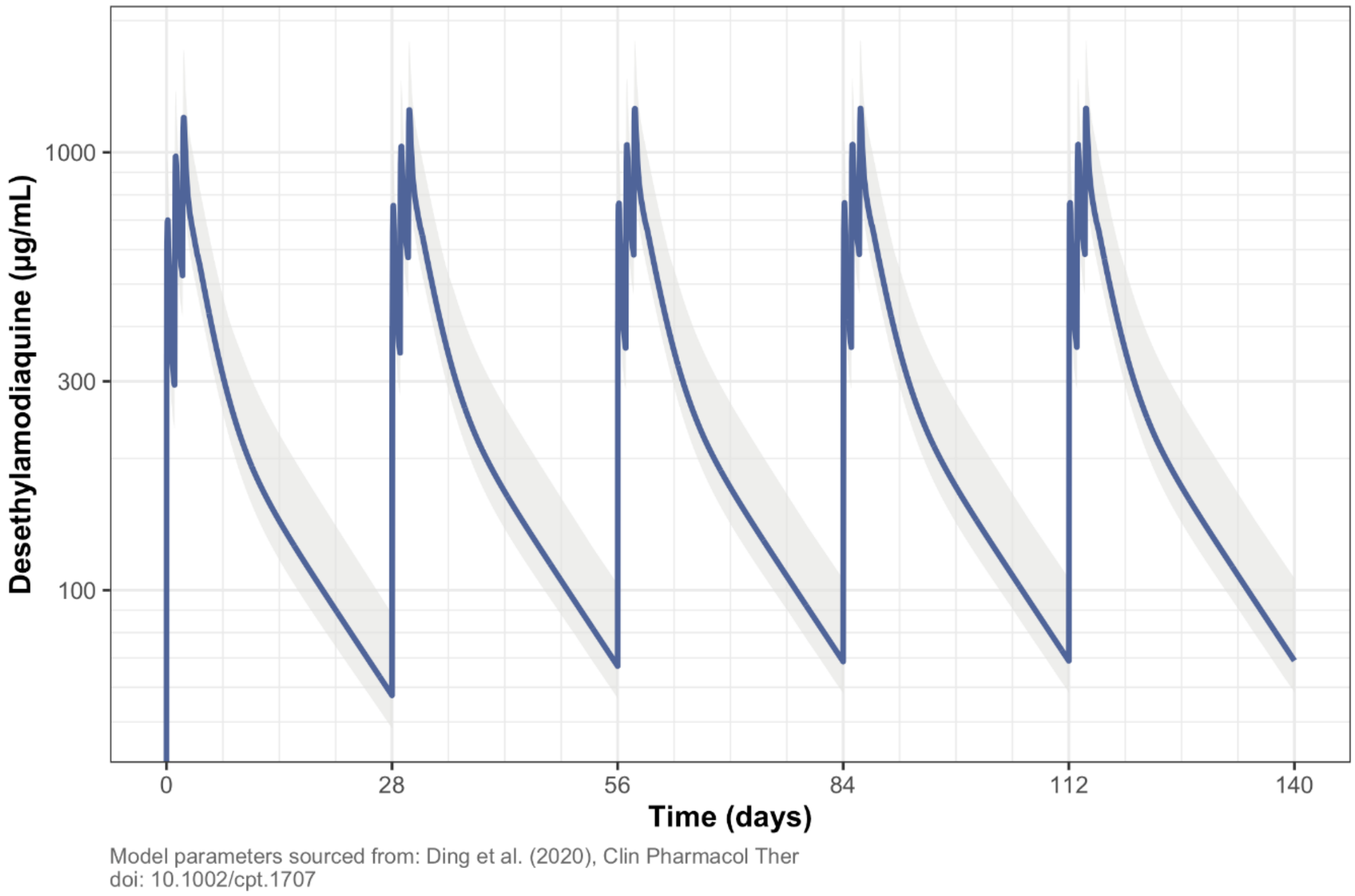

Figure S15: Simulated desethylamodiaquine (DAQ) concentration-time profiles across five consecutive SMC cycles (n = 100 virtual children). Pharmacokinetic simulation of plasma AQ and DAQ concentrations in 100 virtual children aged 3–59 months over three SMC cycles, using a published two-compartment AQ / three-compartment DAQ population pharmacokinetic model [1]. Individual doses were weight-adjusted (75 mg for children aged <12 months; 150 mg for children aged ≥12 months), administered once daily for three consecutive days per cycle. Model parameters: (AQ) CL=101.0 L/h, Vc=314.0 L, Ka=2.85 h^{-1}, Q=119.0 L/h, Vp=1820.0 L; (DAQ) CL=2.33 L/h, Vc=49.1 L, Q1=2.31 L/h, Vp1=363.0 L, Q2=4.34 L/h, Vp2=98.1 L; all parameters allometrically scaled by weight and adjusted for age-related maturation. Lines represent median concentrations across 100 simulated individuals; shaded areas represent the 2.5th–97.5th percentile range.

References

1. Ding, J., et al., *Adherence and Population Pharmacokinetic Properties of Amodiaquine When Used for Seasonal Malaria Chemoprevention in African Children.* Clinical Pharmacology & Therapeutics, 2020. **107**(5): p. 1179-1188.
