# Supplementary material for: Parasitological efficacy of sulfadoxine-pyrimethamine and amodiaquine for seasonal malaria chemoprevention in Africa
Source: Nat Commun. 2026 Oct 1;17:11263. doi: 10.1038/s41467-026-78189-2 (PMC13720088; doi:10.1038/s41467-026-78189-2)
Supplement: Supplementary file 2 [file 41467_2026_78189_MOESM2_ESM.pdf]

Corresponding author(s): Chayanin Sararat

Last updated by author(s): 2026/09/04

Reporting Summary

Nature Portfolio wishes to improve the reproducibility of the work that we publish. This form provides structure for consistency and transparency in reporting. For further information on Nature Portfolio policies, see our Editorial Policies and the Editorial Policy Checklist.

Statistics

For all statistical analyses, confirm that the following items are present in the figure legend, table legend, main text, or Methods section.

n/a	Confirmed	
☐	☒	The exact sample size (*n*) for each experimental group/condition, given as a discrete number and unit of measurement
☐	☒	A statement on whether measurements were taken from distinct samples or whether the same sample was measured repeatedly
☐	☒	The statistical test(s) used AND whether they are one- or two-sided<br>*Only common tests should be described solely by name; describe more complex techniques in the Methods section.*
☐	☒	A description of all covariates tested
☐	☒	A description of any assumptions or corrections, such as tests of normality and adjustment for multiple comparisons
☐	☒	A full description of the statistical parameters including central tendency (e.g. means) or other basic estimates (e.g. regression coefficient) AND variation (e.g. standard deviation) or associated estimates of uncertainty (e.g. confidence intervals)
☐	☒	For null hypothesis testing, the test statistic (e.g. *F*, *t*, *r*) with confidence intervals, effect sizes, degrees of freedom and *P* value noted<br>*Give P values as exact values whenever suitable.*
☒	☐	For Bayesian analysis, information on the choice of priors and Markov chain Monte Carlo settings
☐	☒	For hierarchical and complex designs, identification of the appropriate level for tests and full reporting of outcomes
☒	☐	Estimates of effect sizes (e.g. Cohen's *d*, Pearson's *r*), indicating how they were calculated

Our web collection on statistics for biologists contains articles on many of the points above.

Software and code

Policy information about availability of computer code

Data collection	No custom software was used for data collection. Data were collected using standardised case report forms across the three study sites.
Data analysis	All statistical analyses were conducted in R (version 4.4.3). Key packages: mgcv (version 1.9-3) for generalised additive models; survival (version 3.8-3) for Tobit regression; ipw (version 1.2.2) and survey (version 4.4-2) for inverse probability weighting; ggplot2 (version 3.5.1) for visualisation.<br>All analysis code is available on GitHub (https://github.com/Chayaninss/SMC-Pooled-analysis) and Zenodo (DOI: https://doi.org/10.5281/zenodo.22114310).

For manuscripts utilizing custom algorithms or software that are central to the research but not yet described in published literature, software must be made available to editors and reviewers. We strongly encourage code deposition in a community repository (e.g. GitHub). See the Nature Portfolio guidelines for submitting code & software for further information.

Data

Policy information about availability of data

All manuscripts must include a data availability statement. This statement should provide the following information, where applicable:

- Accession codes, unique identifiers, or web links for publicly available datasets
- A description of any restrictions on data availability
- For clinical datasets or third party data, please ensure that the statement adheres to our policy

The individual participant data used in this analysis are available for access via the WWARN website (https://www.iddo.org/wwarn/accessing-data). Requests for access will be reviewed by a data access committee to ensure that use of data protects the interests of participants and researchers according to the terms of ethics approvals and the principles of equitable data sharing. Requests can be submitted by email to malariaDAC@iddo.org via the data access form. Decisions on access requests are typically communicated within 6–8 weeks of submission.

Research involving human participants, their data, or biological material

Policy information about studies with human participants or human data. See also policy information about sex, gender (identity/presentation), and sexual orientation and race, ethnicity and racism.

Reporting on sex and gender	Sex was recorded at enrolment as a biological variable. The study enrolled both male and female children aged 3–59 months. Sex was not identified as a significant effect modifier in the primary analyses and results are presented for the combined population.
Reporting on race, ethnicity, or other socially relevant groupings	This study was conducted across three sub-Saharan African countries (Uganda, Mozambique, and Burkina Faso). Ethnicity and race were not recorded as variables in the original studies. Study site was included as a covariate in all analyses to account for between-population differences.
Population characteristics	Participants were children aged 3–59 months residing in SMC-eligible areas in Uganda, Mozambique, and Burkina Faso. Children were enrolled at the time of SMC administration and were eligible if they had no contraindications to SPAQ. Key baseline characteristics including age, sex, weight, nutritional status (MUAC), and malaria infection status are summarised in Table 1.
Recruitment	Children were recruited from communities receiving SMC as part of routine implementation programmes. Recruitment was conducted by trained field workers at the time of SMC administration. Written informed consent was obtained from parents or carers prior to enrolment. Inclusion and exclusion criteria are described in the original study publications.
Ethics oversight	Ethical approval was obtained from the relevant institutional review boards at each study site: the Mbale Regional Referral Hospital-REC and Uganda National Council for Science and Technology (Uganda); the Comité Nacional de Bioética para Saúde and Research Ethics Committee of the Hospital Clinic of Barcelona (Mozambique); and the Comité d'Éthique pour la Recherche en Santé (Burkina Faso). All parents or carers provided fully informed written consent prior to enrolment.

Note that full information on the approval of the study protocol must also be provided in the manuscript.

Field-specific reporting

Please select the one below that is the best fit for your research. If you are not sure, read the appropriate sections before making your selection.

☒ Life sciences ☐ Behavioural & social sciences ☐ Ecological, evolutionary & environmental sciences

For a reference copy of the document with all sections, see nature.com/documents/nr-reporting-summary-flat.pdf

Life sciences study design

All studies must disclose on these points even when the disclosure is negative.

Sample size	No formal sample size calculation was performed for this pooled analysis, as it used all available individual participant data from three existing observational studies. The combined analysis population comprised 934 children with complete data at all three timepoints (day 0, day 7, and day 28).
Data exclusions	Children were excluded from the primary analysis if they had missing blood samples at any scheduled timepoint (day 0, day 7, or day 28), if concentrations suggested a repeated dose (amodiaquine >10 ng/mL at day 28, or day 28 > day 7), or if drug concentration z-scores exceeded pre-specified thresholds for extreme values. Potential selection bias from these exclusions is acknowledged in the Discussion. Inverse probability weighting was used in sensitivity analyses to address residual confounding from pharmacokinetic variability.
Replication	This is a pooled analysis of three independent observational studies conducted across three geographically distinct sites in sub-Saharan Africa (Uganda, Mozambique, and Burkina Faso), representing an internal replication across settings with differing transmission intensities and resistance profiles.
Randomization	Randomization was not applicable to this pooled pharmacokinetic–efficacy analysis, which used observational data collected within three SMC

Randomization	implementation studies.
Blinding	Blinding was not applicable. Laboratory qPCR analyses were performed according to standard protocols, with operators unaware of drug concentration results.

Reporting for specific materials, systems and methods

We require information from authors about some types of materials, experimental systems and methods used in many studies. Here, indicate whether each material, system or method listed is relevant to your study. If you are not sure if a list item applies to your research, read the appropriate section before selecting a response.

Materials & experimental systems

n/a	Involved in the study
☒	☐ Antibodies
☒	☐ Eukaryotic cell lines
☒	☐ Palaeontology and archaeology
☒	☐ Animals and other organisms
☐	☒ Clinical data
☒	☐ Dual use research of concern
☒	☐ Plants

Methods

n/a	Involved in the study
☒	☐ ChIP-seq
☒	☐ Flow cytometry
☒	☐ MRI-based neuroimaging

Clinical data

Policy information about clinical studies

All manuscripts should comply with the ICMJE guidelines for publication of clinical research and a completed CONSORT checklist must be included with all submissions.

Clinical trial registration	This manuscript reports a secondary pooled analysis of individual participant data from three registered clinical studies: Uganda (NCT05323721), Mozambique (NCT05186363), and Burkina Faso (NCT05478954). Study designs have been described in the original publications [refs 8, 9].
Study protocol	The study protocols for the Uganda and Mozambique components have been described in detail in the original publications. The Burkina Faso protocol followed the same standardised design.
Data collection	Data were collected prospectively across the three study sites using standardised case report forms. Blood samples were collected at day 0 (pre-dose), day 7, and day 28 for pharmacokinetic and parasitological assessments.
Outcomes	The primary outcome was malaria infection by day 28, defined as qPCR-detectable parasitaemia at day 28 or any episode of intercurrent clinical malaria between day 0 and day 28. The secondary outcome was day 28 cycle threshold (Ct) value from qPCR, used as a continuous measure of parasite density.

Plants

Seed stocks	n/a
Novel plant genotypes	n/a
Authentication	n/a
