# Supplementary material for: Parasitological efficacy of sulfadoxine-pyrimethamine and amodiaquine for seasonal malaria chemoprevention in Africa
Source: Nat Commun. 2026 Oct 1;17:11263. doi: 10.1038/s41467-026-78189-2 (PMC13720088; doi:10.1038/s41467-026-78189-2)
Supplement: Supplementary file 3 [file 41467_2026_78189_MOESM3_ESM.pdf]

nature portfolio

Peer Review File

Parasitological efficacy of sulfadoxine-pyrimethamine and amodiaquine for Seasonal Malaria Chemoprevention in Africa

Corresponding Author: Dr Chayanin Sararat

This file contains all reviewer reports in order by version, followed by all author rebuttals in order by version.

Version 0:

Reviewer comments:

Reviewer #1

(Remarks to the Author)
This large study assesses relationships between plasma drug concentrations and risk of detecting of Plasmodium DNA by qPCR in blood samples obtained 28 days after children <5 years of age received seasonal malaria chemoprevention with SPAQ in 3 different countries representing different African regions with different malaria drug resistance patterns. As millions of doses of SMC are administered to children in multiple countries in Africa to prevent malaria, understanding relationships between drug exposure and malaria prevention is critical. The diversity in representation of individuals from different countries is a strength. The methods are consistent with the best practices of the field. The authors did a good job describing both the results and limitations in the data. This is a well written manuscript with strong methods. I only had a few relatively minor comments below.
1. Although numbers of clinical malaria cases were low, would the authors be able to share summary data on the 7- and 28-day levels of antimalarial for these individuals? It is interesting that the Uganda cohort had a similar number of clinical infections despite having directly observed therapy for all AQ doses. Understanding the drug concentrations among cases would be quite interesting.
2. Would it be possible to share at some point in the manuscript which proportion of the DOT cohort from Uganda that was above the 24 ng/mL desethylamodiaquine level and what proportion in the only first dose observed was in the higher concentration group?
3. qPCR results could remain positive for some time after treatment (e.g. initial parasite positive individuals), can the authors comment on any role this may have in the results, given the high CT values observed in the study.
4. Some of the supplemental figure references in the main text do not align with the figures, please double check.
5. Line 109: can the authors comment on mixed species Plasmodium infections, from figure S2, the proportions of Po and Pf appear to add up to more than 100%. I imagine this is due to mixed infections.
6. Table 2: For the CT values section, can the “N” be included? Are the values only representative of the positive qPCR results or do the summary statistics also represent negatives that were set as a CT value of 40 per the methods? If just the 28 day positive values, the CT values appear quite high suggesting very low prevalence infections.
7. Line 289: Is protective efficacy here referring to clinical disease or qPCR positive?

(Remarks on code availability)

Reviewer #2

(Remarks to the Author)
The authors describe in this paper the Parasitological efficacy of sulfadoxine-pyrimethamine and amodiaquine for Seasonal Malaria Chemoprevention (SMC) in Africa by Sararat et al..,… This is an important topic with regard to the malaria control and elimination efforts. The strategy has saved the life of millions of children in Africa, therefore, the importance to understand the efficacy of the drug employed in the strategy. The manuscript is well written and to the point. However, I have few minor comments that I wish the authors to address as follows:
Line 79-80: restricted the analysis population to children with blood samples available at all three time points (days 0, 7 and 28)…..This denies the readers to understand the average drug concentration for all the participants who reached at D7. It would be proper to include them in the analysis for D7, but when associating with treatment outcome on D28 those present at D7 but not at D28 would be censored. I suggest this to be presented if possible.

Figure1: Flow of participants…..the chart should also include the details for those reached at D7.
Line 97:…..change Plasmodium falciparum to P. falciparum.
Line 108:….change Plasmodium ovale to P. ovale
Line 109:…..change Plasmodium malariae to P. malariae

(Remarks on code availability)

Reviewer #3

(Remarks to the Author)
The present manuscript describes a partial retrospective analysis of three SMC trials in children up to 5-year-old, performed in three African locations. The main objective was a better understanding of the individual contributions of each of the components of the used combination, Sulfadoxine-Pyrimethamine-Amodiaquine (SP-AQ).
In my view, two conclusions are of practical importance, represent the core new message of the work:
(a) DEAQ is the main driver for protection, challenging the importance of the SP component for malaria protection.
(b) DEAQ and Sulfadoxine antagonistic interactions are suggested by the data.

Main comments
The analysis is limited to the first dose of SP-AQ. As such, effects deriving from the "real life" cumulative monthly doses are not considered. This is a shortcoming, as we do not know if the steady state of drugs – namely, of desethylamodiaquine (DEAQ) - is reached along the SMC protocol, including a possible partial offset of the perceived less protective importance of SP; is DEAQ particularly important (or SP particularly irrelevant) during the first month, but becomes less of a dominant player throughout the repetitive SMC exposures? The authors should acknowledge this weakness of the work and provide a detailed justification for why the conclusions can be extrapolated to a complete (i.e. multi-month) procedure.

The cross-site variation in DEAQ levels was interpreted by the authors as the probable result of compliance issues, as the procedure demands a 3-day regimen. The intervention was as such DOT, which constitutes a limitation of the study. The same cannot be claimed for pyrimethamine, part of the 1-dose SP, which also shows significant differences between sites, Uganda showing clearly higher levels (with DEAQ actually matching this pattern when considering D7). I recommend a more thorough discussion, possibly including the possibility of specific population pharmacogenetics effects.

The claim of antagonistic effect between sulfadoxine and amodiaquine is particularly important. It might explain part of the inefficacy of sulfadoxine , in case this interaction is not balanced, affecting the performance of the latter more. I would also be of value to interpret/discuss this data in light of previous works, namely studies performing comparative analysis of pharmacokinetic parameters of SP alone vs SP/Artesunate and SP/AQ (1). Also, this antagonism finding should be more clearly showcased, for example, plotting the D28 CT values vs different proportions of [AQ]/[PYR], which could be used for comparing the parasites from Uganda and Mozambique, carrying the pfdhps K540 allele vs the ones from Burkina Faso without this mutation, to have a first glimpse of a potential contribution of this mutation.
Finally, the importance of this conclusion for the future and potential optimization of the SP + AQ combination demands the confirmation of these observations through a highly controlled in vitro system, e.g., through SP/DEAQ isobologram-based approaches, using parasites with different pfdhps genotypes, in order to understand the influence of these mutations on these antagonistic events.

More information about the PCR and Q-PCR-based methods is needed, specifically concerning the expected (or actually measured during the procedures) limits of detection of each of the methods in terms of the number of parasites per µL. It is also important to note that such methods will not readily distinguish live from dead parasites; parasite DNA has been documented to be present in circulation for weeks after the death of the organism (e.g. (2)).

(2) Haanshuus CG, Mørch K. Detection of remaining Plasmodium DNA and gametocytes during follow up after curative malaria treatment among returned travellers in Norway. Malar J. 2020 Aug 19;19(1):296. doi: 10.1186/s12936-020-03367-6. PMID: 32814587; PMCID: PMC7436973.

(1) Tekete MM, Toure S, Fredericks A, Beavogui AH, Sangare CP, Evans A, Smith P, Maiga H, Traore ZI, Doumbo OK, Barnes KI, Djimde AA. Effects of amodiaquine and artesunate on sulphadoxine-pyrimethamine pharmacokinetic parameters in children under five in Mali. Malar J. 2011 Sep 21;10:275. doi: 10.1186/1475-2875-10-275. PMID: 21936885; PMCID: PMC3197573.

Other comments
I would like to suggest that the authors add a figure with the flux of analysis, as the text can be confusing. In the "Malaria infection prevalence" section, the initial total number of infections has an n = 438, then referring a n = 478, which is the number including the intercurrent clinical malaria cases happening during follow-up. This number is only relatively clarified later in the text.

Some information concerning the transmission characteristics of each one of the sites would be welcoming, in order to better understand how diverse (or not) these sites are, as analysis lumping the data of the three of them is performed in some section of this report.

One is left wondering if the “small number of outliers” with higher levels of amodiaquine at D28 - a time where in fact, AQ is not expected to be generally detectable - correspond to carriers of defective CYP2C8 alleles (3). This is data easy to generate and would enrich the article, in tandem with considerations on the markedly higher pyrimethamine drug levels observed in Uganda at both D7 and D28 time points.

(3) Parikh S, Ouedraogo JB, Goldstein JA, Rosenthal PJ, Kroetz DL. Amodiaquine metabolism is impaired by common polymorphisms in CYP2C8: implications for malaria treatment in Africa. Clin Pharmacol Ther. 2007 Aug;82(2):197-203. doi: 10.1038/sj.clpt.6100122. Epub 2007 Mar 14. PMID: 17361129.

SP is known to have important positive effects beyond the simple protection against malaria parasites, at least in the context of IPTp, due to inherent broader anti-microbial activity. This aspect is very important for a complete evaluation of the importance of SP as a SMC partner, and should be taken in consideration in the discussion of the potential implications of the presented data.

Where is the data related to the pfaat gene?

(Remarks on code availability)

Open Access This Peer Review File is licensed under a Creative Commons Attribution 4.0 International License, which permits use, sharing, adaptation, distribution and reproduction in any medium or format, as long as you give appropriate credit to the original author(s) and the source, provide a link to the Creative Commons license, and indicate if changes were made.
In cases where reviewers are anonymous, credit should be given to 'Anonymous Referee' and the source.
The images or other third party material in this Peer Review File are included in the article’s Creative Commons license, unless indicated otherwise in a credit line to the material. If material is not included in the article’s Creative Commons license and your intended use is not permitted by statutory regulation or exceeds the permitted use, you will need to obtain permission directly from the copyright holder.
To view a copy of this license, visit https://creativecommons.org/licenses/by/4.0/

Response to Referee Comments

Reviewer #1

"This large study assesses relationships between plasma drug concentrations and risk of detecting of Plasmodium DNA by qPCR in blood samples obtained 28 days after children <5 years of age received seasonal malaria chemoprevention with SPAQ in 3 different countries representing different African regions with different malaria drug resistance patterns. As millions of doses of SMC are administered to children in multiple countries in Africa to prevent malaria, understanding relationships between drug exposure and malaria prevention is critical. The diversity in representation of individuals from different countries is a strength. The methods are consistent with the best practices of the field. The authors did a good job describing both the results and limitations in the data. This is a well written manuscript with strong methods. I only had a few relatively minor comments below."

Response:
We thank you for your careful review and generous assessment of our work. We have addressed each of your comments below.

"1. Although numbers of clinical malaria cases were low, would the authors be able to share summary data on the 7- and 28-day levels of antimalarial for these individuals?

It is interesting that the Uganda cohort had a similar number of clinical infections despite having directly observed therapy for all AQ doses. Understanding the drug concentrations among cases would be quite interesting."

Response:
We thank you for this observation. We have added a supplementary figure showing boxplots of drug concentrations comparing children who experienced intercurrent clinical malaria during follow-up against those who did not (Figure S5). We have noted this observation briefly in the Results, lines 137-138. In addition, we fitted the same multivariable models to the outcome of intercurrent malaria and see that higher day 28 desethylamodiaquine was the only drug exposure significantly associated with reduced risk of intercurrent malaria (OR = 0.49, 95%CI 0.27-0.87, p-value 0.02).

Drug concentrations among children who experienced intercurrent clinical malaria during follow-up are summarised in Figure S5.

"2. Would it be possible to share at some point in the manuscript which proportion of the DOT cohort from Uganda that was above the 24 ng/mL desethylamodiaquine level and what proportion in the only first dose observed was in the higher concentration group?"

Response:

Thank you for this suggestion. All children in Uganda had DOT for all 3 amodiaquine doses whereas this was not the case for Mozambique or Burkina Faso. We have added to line 172-173:

In Uganda, 63% (255/407) had day 28 desethylamodiaquine >24 ng/mL, whereas in Burkina Faso this was 55% (232/423) and in Mozambique 24% (25/104).

"3. qPCR results could remain positive for some time after treatment (e.g. initial parasite positive individuals), can the authors comment on any role this may have in the results, given the high CT values observed in the study."

Response:

We thank you for raising this point, which is also mentioned by another reviewer. We agree that residual parasite DNA might contribute to PCR positivity. We consider this unlikely to greatly affect our findings. We compared day-28 parasite densities estimated by qPCR against those concurrently measured by microscopy. The two measures show consistent agreement at high parasite densities with discordance limited to low parasite densities ($<10^4$ parasite per mL) where microscopy is known to have reduced sensitivity. Furthermore, a sensitivity analysis treating day 28 Ct values above 35 as negative, thereby excluding low-density infections, yielded consistent exposure-response relationships for all three drug components (Supplementary Results), further supporting the robustness of our primary findings. We have added this comparison as supplementary figure (Figure S14) and added a section in the discussion, lines 290-298.

Third, PCR-based detection cannot readily distinguish live from non-viable parasites, as parasite DNA can theoretically persist in blood. Parasite DNA persistence would result in contamination of the PCR based outcome, thus flattening the exposure-response curve. We think it is highly unlikely that DNA persistence has materially affected our findings for two reasons. The day-28 PCR estimated parasite densities agree closely with concurrent microscopy based estimates in samples from Uganda (Figure S14). In addition, when we use a more stringent Ct value cutoff of 35 cycles (excluding low density infections or false positive results) we obtain near identical exposure response relationships (Supplementary Results).

Sensitivity analysis: alternative Ct threshold for PCR positivity

To access the robustness of the primary analysis to the choices of Ct threshold for defining PCR positivity, we repeated the multivariable logistic regression using stricter threshold of Ct < 35 as the definition of a positive result, thereby excluding low-density infections that may represent residual non-viable parasite DNA. The estimated exposure-response relationships were consistence with the primary analysis for all three drug components: desethylamodiaquine OR = 0.46 (95% CI 0.36–0.58), sulfadoxine OR = 0.84 (95% CI 0.65–1.07), and pyrimethamine OR = 0.86 (95% CI 0.65–1.13), compared with OR = 0.55 (95% CI 0.44–0.68), OR = 0.80 (95% CI 0.63–1.00), and OR = 0.88 (95% CI 0.67–1.20) in the primary analysis, respectively.

"4. Some of the supplemental figure references in the main text do not align with the figures, please double check."

Response:
We thank you for catching this. We have carefully cross-checked all supplementary figure references.

"5. Line 109: can the authors comment on mixed species Plasmodium infections, from figure S2, the proportions of Po and Pf appear to add up to more than 100%. I imagine this is due to mixed infections."

Response:
We thank you for the observation and confirm your interpretation is correct. We have clarified this in the text and Figure S3 caption.

"6. Table 2: For the CT values section, can the "N" be included? Are the values only representative of the positive qPCR results or do the summary statistics also represent negatives that were set as a CT value of 40 per the methods? If just the 28 day positive values, the CT values appear quite high suggesting very low prevalence infections."

Response:
We thank you for this observation. We have revised Table 2 to include the number of PCR-positive and PCR-negative samples for each site, and have recalculated the Ct summary statistics restricted to PCR-positive samples only.

Table 1: Summary of efficacy outcomes by country.

Site	Uganda	Mozambique	Burkina Faso
Malaria infection (n, %)*	125/407 (31%)	45/104 (43%)	140/423 (33%)
PCR positive on day 28 (n, %)	109/407 (27%)	44/104 (42%)	131/423 (31%)
Intercurrent clinical malaria* (n, %)	24/407 (6%)	3/104 (3%)	17/423 (4%)
**Cycle threshold (Ct) values**			
N (Ct = 40, PCT negative)	298	60	292
N (Ct < 40, PCT positive)	109	44	131
Range (min-max), Ct < 40 only	18.7-38.6	19.8-37.9	18.8-38.9
Mean, Ct < 40 only	33.8	32.6	31.9
Median (IQR), Ct < 40 only	35 (32.5-36.1)	34 (31.2-35.9)	34 (29.5-35.4)

*Malaria infection is defined at PCR positive by day 28 or intercurrent malaria illness (fever or history of fever within 24 hours with a positive RDT).

“7. Line 289: Is protective efficacy here referring to clinical disease or qPCR positive?”

Response:

We thank you for this important clarification request. We have clarified that “protective efficacy” refers to the primary outcome (qPCR-detectable parasitaemia or intercurrent clinical malaria), as defined in the Methods.

Discussion, lines 256-258

Given that desethylamodiaquine concentrations are below optimal for many children even after fully observed treatment, this implies that amodiaquine alone cannot achieve very high protective efficacy against malaria infection, defined as PCR-detectable parasitaemia or intercurrent clinical malaria.

Reviewer #2

"The authors describe in this paper the Parasitological efficacy of sulfadoxine-pyrimethamine and amodiaquine for Seasonal Malaria Chemoprevention (SMC) in Africa by Sararat et al..,… This is an important topic with regard to the malaria control and elimination efforts. The strategy has saved the life of millions of children in Africa, therefore, the importance to understand the efficacy of the drug employed in the strategy. The manuscript is well written and to the point. However, I have few minor comments that I wish the authors to address as follows:"

Response:
We thank you for your careful review and positive assessment of our work. We have addressed each of your comments below.

"Line 79-80: restricted the analysis population to children with blood samples available at all three time points (days 0, 7 and 28)…..This denies the readers to understand the average drug concentration for all the participants who reached at D7. It would be proper to include them in the analysis for D7, but when associating with treatment outcome on D28 those present at D7 but not at D28 would be censored. I suggest this to be presented if possible."

Response:
We thank you for this helpful suggestion. We have extended table S2 to include summary of antimalarial concentrations across the entire available population.

As the qPCR-confirmed outcome was only available at day 28, children missing this timepoint could not be included in the primary efficacy analysis; this is now clarified in the Methods, line 361-365.

The primary efficacy analysis was restricted to children with blood samples available at all three timepoints (day 0, day 7, day 28), as the qPCR-confirmed malaria outcome was assessed at day 28 and required drug concentration data across the dosing interval for inclusion in the regression model. To provide more complete picture of drug exposure across the study population, descriptive summaries of drug concentrations among all children with an available sample at each timepoint are presented in Table S2.

Table S2: Drug concentration summary (median, IQR) across the efficacy analysis population and full available population.

	Concentration (ng/mL)		
	**Uganda**	**Mozambique**	**Burkina Faso**
**Efficacy analysis population**			
Sulfadoxine (Day 7)	28 (22.2 - 38.7)$\times 10^3$	24.5 (16.1 – 32.7)$\times 10^3$	31.3 (21.0 - 41.5)$\times 10^3$
Sulfadoxine (Day 28)	1.6 (<LLOQ - 3.7)$\times 10^3$	<LLOQ (<LLOQ - 1.7)$\times 10^3$	1.0 (<LLOQ - 3.2)$\times 10^3$
Pyrimethamine (Day 7)	91 (70 - 120)	46 (35 - 70)	49 (34 - 69)
Pyrimethamine (Day 28)	<LLOQ (<LLOQ - 6)	<LLOQ (<LLOQ)	<LLOQ (<LLOQ)

Amodiaquine (Day 7)	2 (<LLOQ - 5)	<LLOQ (<LLOQ - 2)	3 (<LLOQ - 5)
Amodiaquine (Day 28)	<LLOQ	<LLOQ	<LLOQ
Desethylamodiaquine (Day 7)	155 (117 - 205)	99 (69 - 148)	98 (69 - 140)
Desethylamodiaquine (Day 28)	30 (18 - 46)	14 (9 - 24)	26 (17- 39)
**All samples**			
Sulfadoxine (Day 7)	29 (22.3 - 38.5) $\times 10^3$	20.2 (13.7 – 30.5) $\times 10^3$	31.4 (21.0 - 41.4)$\times 10^3$
Sulfadoxine (Day 28)	1.6 <LLOQ - 3.7) $\times 10^3$	<LLOQ (<LLOQ - 1.2)$\times 10^3$	1.1 (<LLOQ - 3.2)$\times 10^3$
Pyrimethamine (Day 7)	92 (70 - 120)	44 (27 - 61)	49 (34 - 68)
Pyrimethamine (Day 28)	<LLOQ (<LLOQ - 6)	<LLOQ (<LLOQ)	<LLOQ (<LLOQ)
Amodiaquine (Day 7)	2 (<LLOQ - 5)	<LLOQ (<LLOQ - 2)	3 (<LLOQ - 5)
Amodiaquine (Day 28)	<LLOQ	<LLOQ	<LLOQ
Desethylamodiaquine (Day 7)	156 (117 - 206)	88 (48 - 130)	98 (68 - 138)
Desethylamodiaquine (Day 28)	30 (18 - 46)	14 (9 - 23)	26 (17- 39)

“Figure1: Flow of participants…..the chart should also include the details for those reached at D7.”

Response:

We thank you for this suggestion. We have revised the CONSORT flow diagram (Figure 1) to distinguish between two analysis populations:

- Descriptive analysis population: all children with samples available at day 0, day 7, or day 28, included for descriptive summaries of drug concentrations at each timepoint
- Pharmacokinetic-efficacy analysis population: children with complete samples at all three timepoints (day 0, day 7, and day 28), used for the primary PK-efficacy analyses

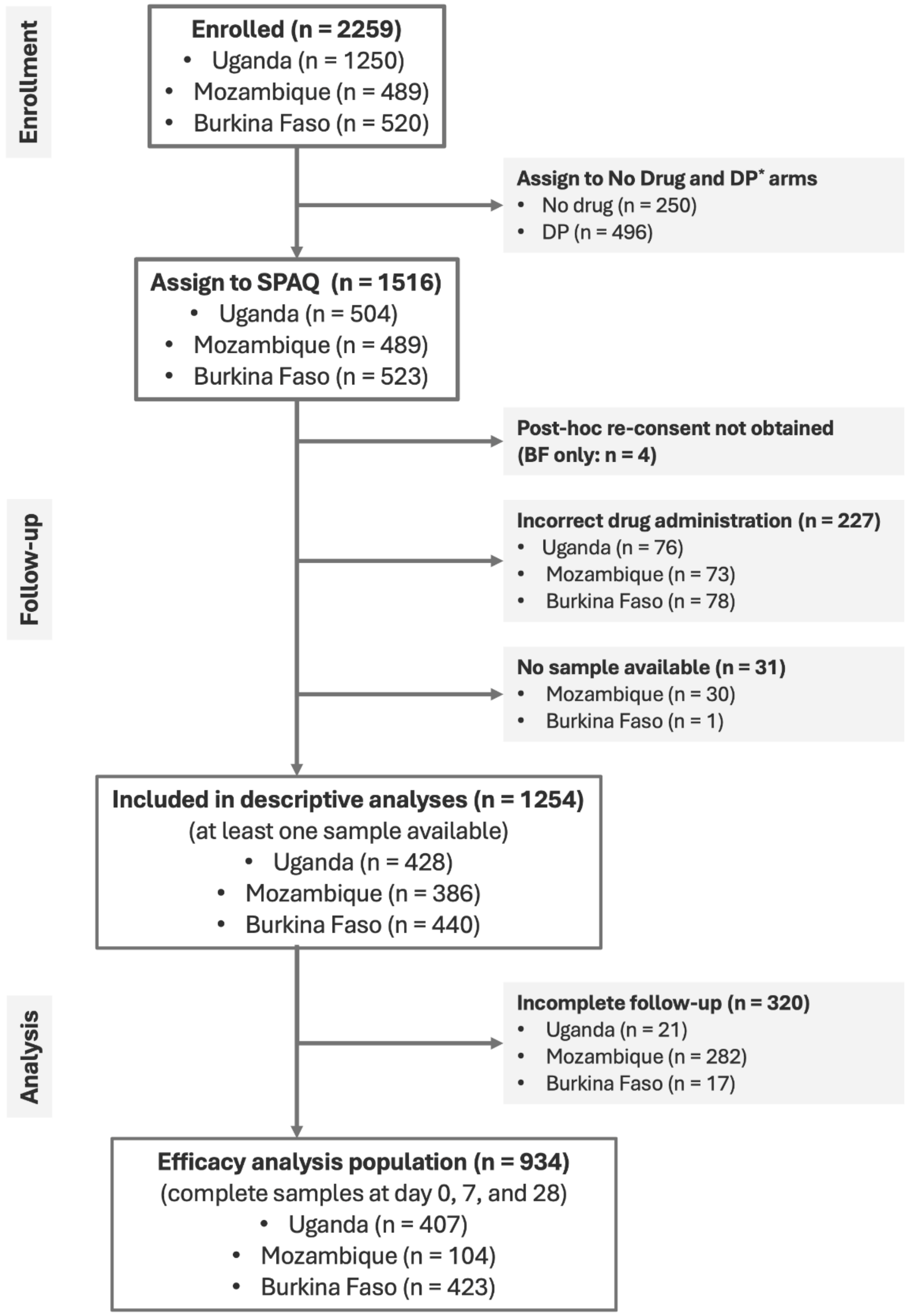

* DP: dihydroartemisinin-piperaquine

Figure 1: Flow of participants through enrolment, exclusion, follow-up, and inclusion in the analysis population across the three study sites.

"Line 97:…..change Plasmodium falciparum to P. falciparum.

Line 108:….change Plasmodium ovale to P. ovale

Line 109:…..change Plasmodium malariae to P. malariae”

Response:

We have corrected all subsequent mentions to use the abbreviated genus name throughout the manuscript.

Reviewer #3

"The present manuscript describes a partial retrospective analysis of three SMC trials in children up to 5-year-old, performed in three African locations. The main objective was a better understanding of the individual contributions of each of the components of the used combination, Sulfadoxine-Pyrimethamine-Amodiaquine (SP-AQ). In my view, two conclusions are of practical importance, represent the core new message of the work:
(a) DEAQ is the main driver for protection, challenging the importance of the SP component for malaria protection.
(b) DEAQ and Sulfadoxine antagonistic interactions are suggested by the data."

Response:
Thank you for your thorough review. We address each of your comments in detail below.

Main comments

"The analysis is limited to the first dose of SP-AQ. As such, effects deriving from the "real life" cumulative monthly doses are not considered. This is a shortcoming, as we do not know if the steady state of drugs – namely, of desethylamodiaquine (DEAQ) - is reached along the SMC protocol, including a possible partial offset of the perceived less protective importance of SP; is DEAQ particularly important (or SP particularly irrelevant) during the first month, but becomes less of a dominant player throughout the repetitive SMC exposures? The authors should acknowledge this weakness of the work and provide a detailed justification for why the conclusions can be extrapolated to a complete (i.e. multi-month) procedure."

Response:
We acknowledge this as a potential limitation. The CPES study was designed to characterise PK-efficacy relationships within a single chemoprevention treatment cycle. We think that these results will generalize well to multi-cycle chemoprevention because there is almost no build-up of sulfadoxine or desethylamodiaquine over multiple cycles.

To show the negligible accumulation of desethylamodiaquine across repeated SMC cycles, we conducted a pharmacokinetic simulation of five consecutive SMC cycles using a published population PK model for amodiaquine and desethylamodiaquine in African children. This simulation supports the interpretation that each SMC cycle functions as a largely pharmacokinetically independent episode. We have updated the Discussion, lines 307-315 accordingly.

Fifth, this analysis was restricted to a single SMC cycle, as multi-cycle individual-level data were not available for pooled analysis. However, efficacy is not expected to vary substantially across cycles because desethylamodiaquine will not accumulate over multiple cycles (see simulated profiles in Figure S15 based on an existing PK model [23]).

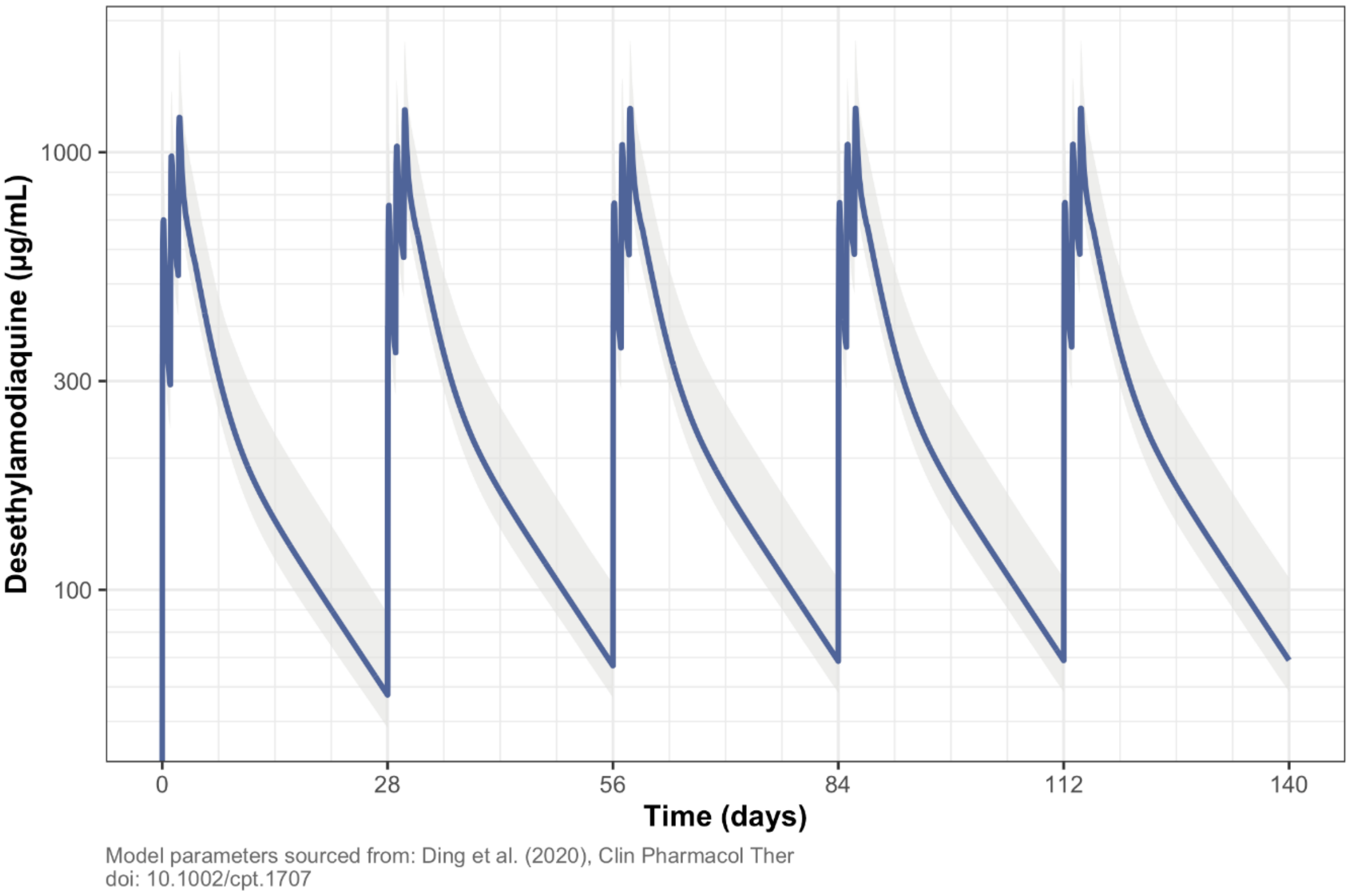

Figure S15: Simulated desethylamodiaquine (DAQ) concentration-time profiles across five consecutive SMC cycles. Pharmacokinetic simulation of plasma AQ and DAQ concentrations in 100 virtual children aged 3–59 months over three SMC cycles, using a published two-compartment AQ / three-compartment DAQ population pharmacokinetic model [11]. Individual doses were weight-adjusted (75 mg for children aged <12 months; 150 mg for children aged ≥12 months), administered once daily for three consecutive days per cycle. Model parameters: (AQ) CL=101.0 L/h, Vc=314.0 L, Ka=2.85 h^{-1}, Q=119.0 L/h, Vp=1820.0 L; (DAQ) CL=2.33 L/h, Vc=49.1 L, Q1=2.31 L/h, Vp1=363.0 L, Q2=4.34 L/h, Vp2=98.1 L; all parameters allometrically scaled by weight and adjusted for age-related maturation. Lines represent median concentrations across 100 simulated individuals; shaded areas represent the 2.5th–97.5th percentile range.

"The cross-site variation in DEAQ levels was interpreted by the authors as the probable result of compliance issues, as the procedure demands a 3-day regimen. The intervention was as such DOT, which constitutes a limitation of the study. The same cannot be claimed for pyrimethamine, part of the 1-dose SP, which also shows significant differences between sites, Uganda showing clearly higher levels (with DEAQ actually matching this pattern when considering D7). I recommend a more thorough discussion, possibly including the possibility of specific population pharmacogenetics effects."

Response:

We thank you for this important observation and we have expanded the Discussion accordingly. It is unclear why there are such substantial differences in pyrimethamine concentrations.

Discussion lines 260-273 (new paragraph added)

The variation in the sulfadoxine-pyrimethamine ratios across sites, whereby sulfadoxine concentrations were broadly comparable but pyrimethamine concentrations were almost twice as high in Uganda, cannot reflect differences in adherence given that both drugs are co-administered as a single tablet. Biological factors, including possible pharmacogenetic differences in drug metabolism might contribute to this inter-site variation. Sulfadoxine undergoes well-characterised hepatic metabolism and is primarily eliminated by renal filtration [4], which might account for its consistent concentrations across sites. In contrast, pyrimethamine is metabolised via hepatic pathways that remain incompletely characterised [19], rendering it potentially more susceptible to inter-population pharmacogenetic variability. For desethylamodiaquine, incomplete adherence to the second and third amodiaquine doses at sites without confirmed directly observed therapy remains the most plausible contributor to inter-site variability. CYP2C8 polymorphisms, prevalent in West African populations [20] and shown in vitro to impair amodiaquine metabolism [21], might represent an additional biological determinant. Prospective CYP genotyping in future SMC pharmacokinetic studies would help disentangle the relative contributions of adherence and pharmacogenetic variation to inter-site differences in drug exposure.

"The claim of antagonistic effect between sulfadoxine and amodiaquine is particularly important. It might explain part of the inefficacy of sulfadoxine, in case this interaction is not balanced, affecting the performance of the latter more. It would also be of value to interpret/discuss this data in light of previous works, namely studies performing comparative analysis of pharmacokinetic parameters of SP alone vs SP/Artesunate and SP/AQ (1).

Also, this antagonism finding should be more clearly showcased, for example, plotting the D28 CT values vs different proportions of [AQ]/[PYR], which could be used for comparing the parasites from Uganda and Mozambique, carrying the pfdhps K540 allele vs the ones from Burkina Faso without this mutation, to have a first glimpse of a potential contribution of this mutation.

(1) Tekete MM, Toure S, Fredericks A, Beavogui AH, Sangare CP, Evans A, Smith P, Maiga H, Traore ZI, Doumbo OK, Barnes KI, Djimde AA. Effects of amodiaquine and artesunate on sulphadoxine-pyrimethamine pharmacokinetic parameters in children under five in Mali. Malar J. 2011 Sep 21;10:275. doi: 10.1186/1475-2875-10-275. PMID: 21936885; PMCID: PMC3197573."

Response:
We thank you for this important suggestion. We have revised the relevant content to describe the observed drug interaction as "concentration-dependent attenuation of the desethylamodiaquine protective effect", avoiding the term "antagonism" to prevent overclaiming an interpretation that our data alone cannot fully support.

We have added a plot of day 28 Ct values against day 28 desethylamodiaquine concentrations, stratified by day 7 sulfadoxine levels (low/medium/high tertiles) (Figure S12). This figure shows a steeper slope at lower sulfadoxine levels, indicating a stronger desethylamodiaquine protective effect.

Regarding the potential contribution of pfdhps K540E, a first glimpse can be gained from Figure 5. The desethylamodiaquine effect on day 28 Ct was broadly comparable across sites, while the effects

of sulfadoxine and pyrimethamine were greater in Burkina Faso, where pfdhps K540E was almost entirely absent. To formally evaluate this observation, we conducted exploratory site-specific interaction analyses in left-censored Tobit models, the results of which are presented in the Supplement Results. Briefly, site-specific estimates suggested a stronger sulfadoxine protective effect in Burkina Faso. The site-specific estimates of the desethylamodiaquine×sulfadoxine attenuation were significant in Uganda and Burkina Faso, with a consistent but non-significant estimate in Mozambique, likely reflecting limited statistical power at that site.

Regarding the suggestion to contextualise our findings within prior SP pharmacokinetic literature, Tekete et al. (2011) demonstrated that co-administration of amodiaquine with SP does not meaningfully alter day 7 sulfadoxine or pyrimethamine concentrations in young Malian children, supporting the interpretation that the concentration-dependent attenuation we observed is likely pharmacodynamic rather than pharmacokinetic, and we have incorporated this context into the revised Discussion.

Discussion Lines 274-276

The concentration-dependent attenuation observed between desethylamodiaquine and sulfadoxine (Figure S12) warrants careful mechanistic interpretation. There does not seem to be a strong pharmacokinetic interaction between amodiaquine and SP [22]. Therefore the observed saturation in effect is likely to be a result of the pharmacodynamics (near maximal inhibition reached). Further in vitro work better characterizing this interaction as a function of the known SP resistance mutations would be useful to know how these results generalise.

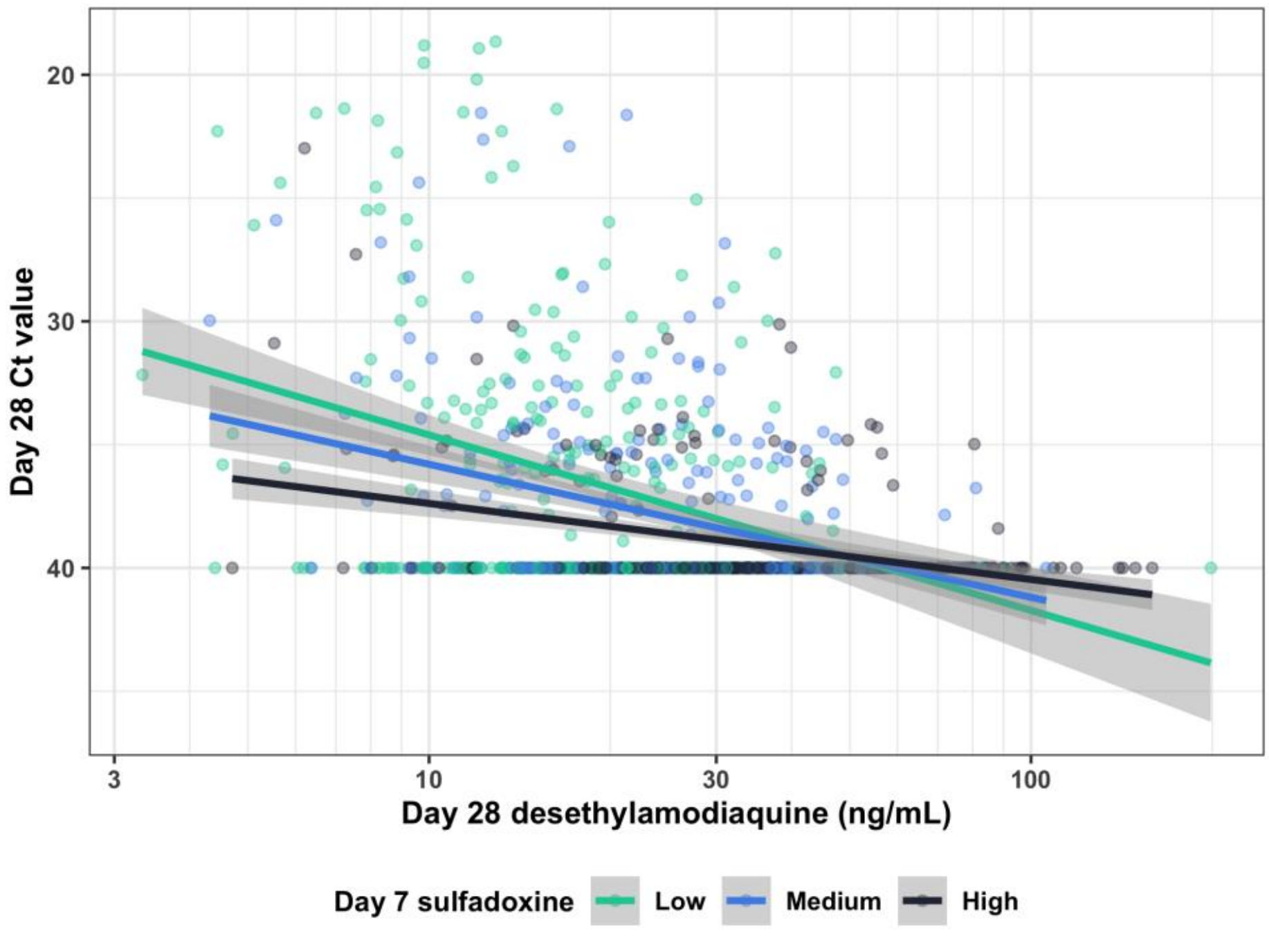

Figure S12: Concentration-dependent attenuation of desethylamodiaquine protective effect by sulfadoxine. Scatter plot of day 28 Ct values against day 28 desethylamodiaquine concentrations, stratified by day 7 sulfadoxine concentration tertiles (low, medium, and high). The y-axis is reversed

such that higher Ct values reflect lower parasitaemia. Lines represent linear regression fits with 95% confidence intervals for each sulfadoxine tertile. A steeper slope in the low sulfadoxine group indicates a stronger desethylamodiaquine protective effect at lower sulfadoxine concentrations.

"Finally, the importance of this conclusion for the future and potential optimization of the SP + AQ combination demands the confirmation of these observations through a highly controlled in vitro system, e.g., through SP/DEAQ isobologram-based approaches, using parasites with different pfdhps genotypes, in order to understand the influence of these mutations on these antagonistic events."

Response:
We agree that in vitro data could shed more light on this interaction between the drugs. However, this is out of scope for this work. We have added this as a suggestion for future work in the Discussion lines 315-319 .

"More information about the PCR and Q-PCR-based methods is needed, specifically concerning the expected (or actually measured during the procedures) limits of detection of each of the methods in terms of the number of parasites per μL. It is also important to note that such methods will not readily distinguish live from dead parasites; parasite DNA has been documented to be present in circulation for weeks after the death of the organism (e.g. (2)).

(2) Haanshuus CG, Mørch K. Detection of remaining Plasmodium DNA and gametocytes during follow up after curative malaria treatment among returned travellers in Norway. Malar J. 2020 Aug 19;19(1):296. doi: 10.1186/s12936-020-03367-6. PMID: 32814587; PMCID: PMC7436973."

Response:
We thank the reviewer for this constructive feedback. We have updated the Methodology section to explicitly state the limits of detection for qPCR assays used in this study (lines 335).

The reviewer raises an excellent point regarding the inherent limitation of DNA-based molecular diagnostics. Because cell-free or residual parasite DNA can persist in the bloodstream for several weeks following successful treatment or natural clearance, positive PCR/qPCR results do not definitively confirm the presence of viable, active infections.

However, to address to this concern, we compared day-28 parasite density by PCR and microscopy. The two methods show consistent trends across the density range, with discordance observed at low parasite densities (~ $<10^4$) where microscopy is known to have limited sensitivity (see response to reviewer 1). A sensitivity analysis was performed treating 28 Ct values above 35 as negative to exclude low-density infections that may represent residual non-viable parasite DNA. The exposure-response relationships were consistent with the primary analysis across all drug components. We have mentioned this limitation in the discussion.

Discussion, lines 290-298:

Third, PCR-based detection cannot readily distinguish live from non-viable parasites, as parasite DNA can theoretically persist in blood. Parasite DNA persistence would result in contamination of

the PCR based outcome, thus flattening the exposure-response curve. We think it is highly unlikely that DNA persistence has materially affected our findings for two reasons. The day-28 PCR estimated parasite densities agree closely with concurrent microscopy based estimates in samples from Uganda (Figure S14). In addition, when we use a more stringent Ct value cutoff of 35 cycles (excluding low density infections or false positive results) we obtain near identical exposure response relationships (Supplementary Results).

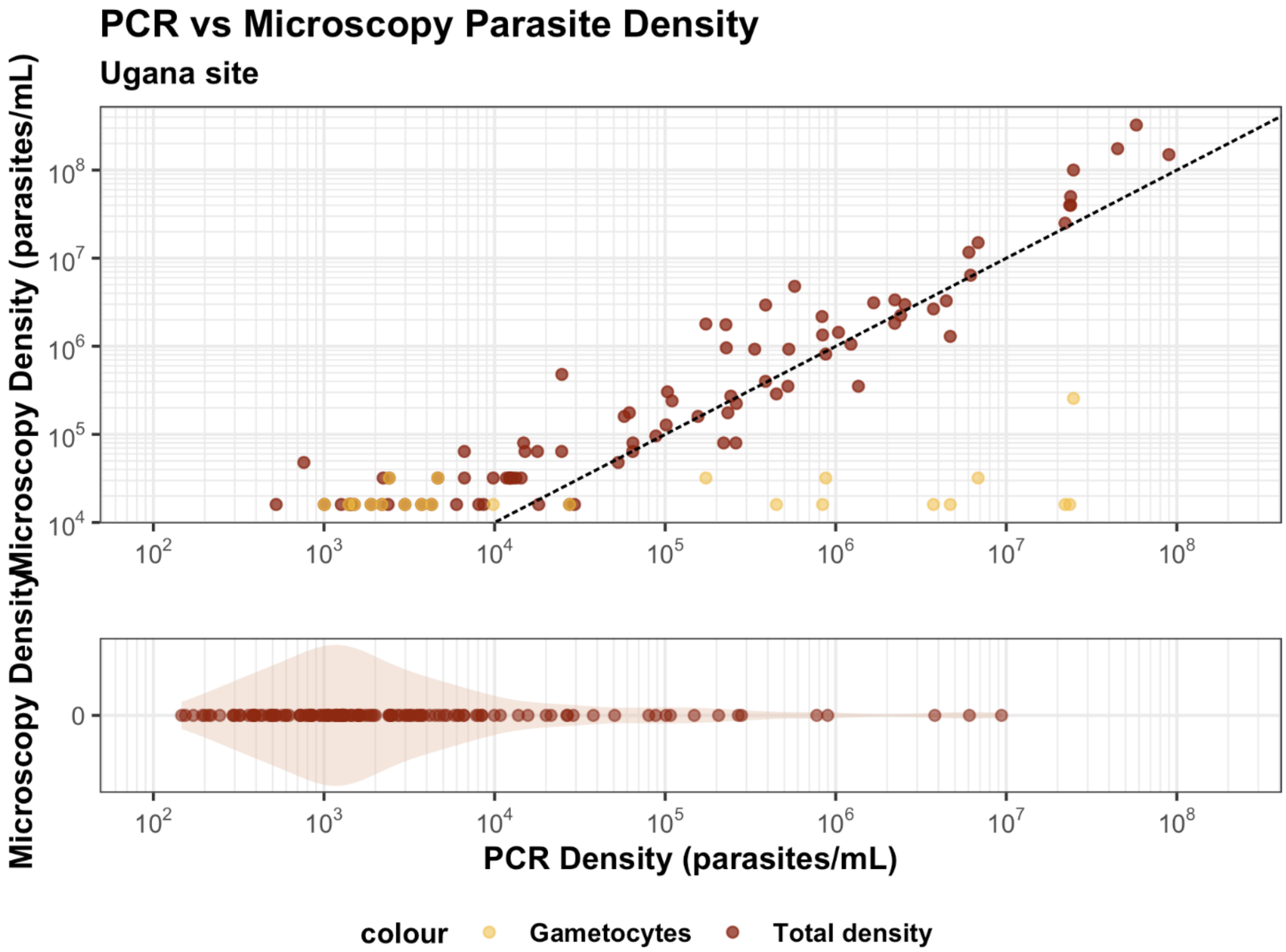

Figure S14: Agreement between PCR-estimated and microscopy-determined parasite densities at day-28. Blood slides were read by microscopy only for samples that were PCR positive. This comparison is restricted to the Uganda site due to limited quality at the other two sites. The two methods showed consistent agreement at high parasite densities with discordance confined to low densities ($<10^4$ parasites/mL) where microscopy is known to have reduced sensitivity.

Sensitivity analysis: alternative Ct threshold for PCR positivity

To access the robustness of the primary analysis to the choices of Ct threshold for defining PCR positivity, we repeated the multivariable logistic regression using stricter threshold of Ct < 35 as the definition of a positive result, thereby excluding low-density infections that may represent residual non-viable parasite DNA. The estimated exposure-response relationships were consistence with the primary analysis for all three drug components: desethylamodiaquine OR = 0.46 (95% CI 0.36–0.58), sulfadoxine OR = 0.84 (95% CI 0.65–1.07), and pyrimethamine OR = 0.86 (95% CI 0.65–1.13), compared with OR = 0.55 (95% CI 0.44–0.68), OR = 0.80 (95% CI 0.63–1.00), and OR = 0.88 (95% CI 0.67–1.20) in the primary analysis, respectively.

“Other comments

I would like to suggest that the authors add a figure with the flux of analysis, as the text can be confusing. In the “Malaria infection prevalence” section, the initial total number of infections has an n = 438, then referring a n = 478, which is the number including the intercurrent clinical malaria cases happening during follow-up. This number is only relatively clarified later in the text.”

Response:

Thank you for this confusion and important suggestion. The number n = 438 was a typographical error. The correct number is n = 478. We have corrected this error and have revised the CONSORT diagram to distinguish descriptive analysis population (all children who had at least one sample available) and efficacy analysis population (all children with complete follow-up).

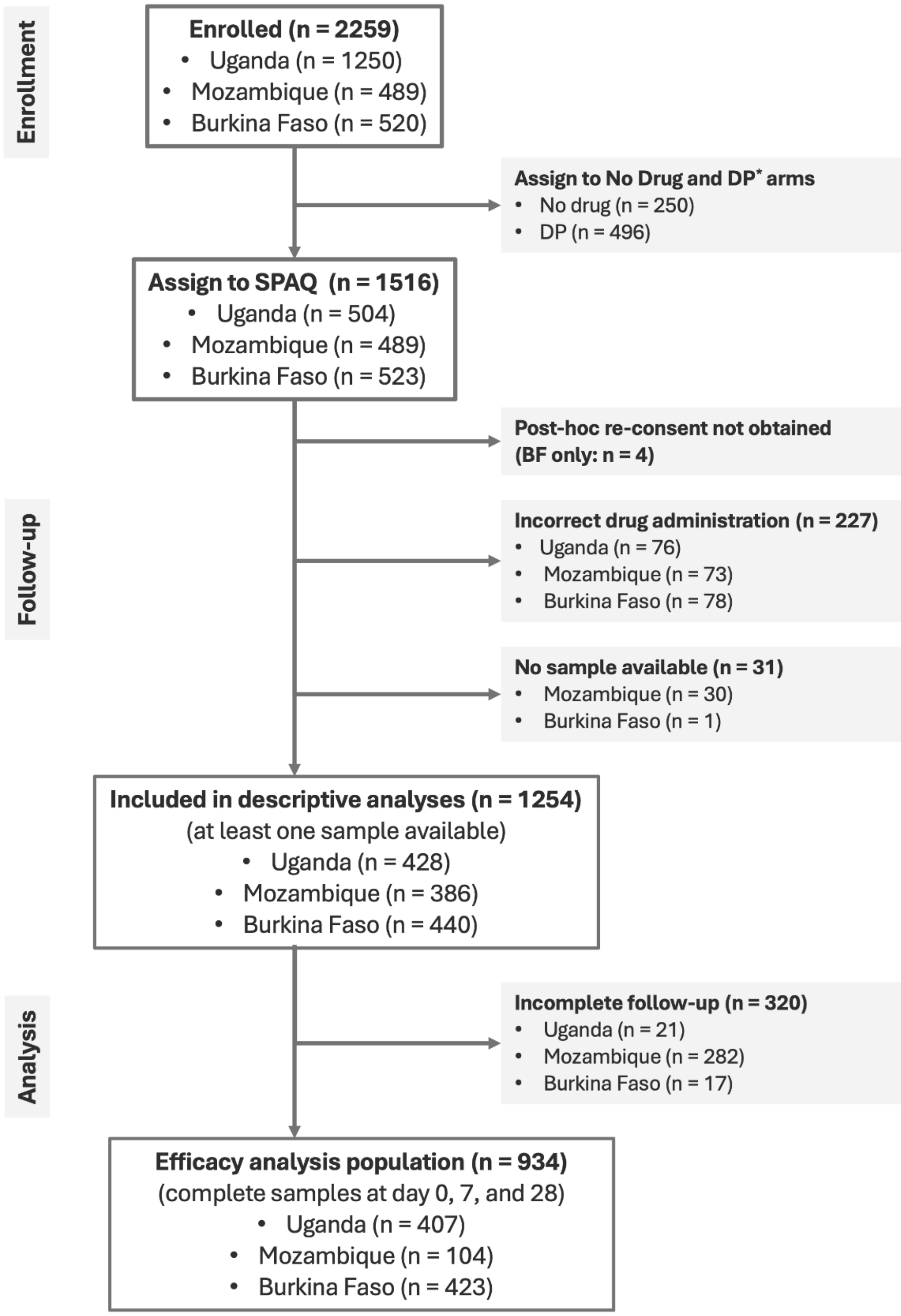

* DP: dihydroartemisinin-piperaquine

Figure 1: Flow of participants through enrolment, exclusion, follow-up, and inclusion in the analysis population across the three study sites.

“Some information concerning the transmission characteristics of each one of the sites would be welcoming, in order to better understand how diverse (or not) these sites are, as analysis lumping the data of the three of them is performed in some section of this report.”

Response:
We thank you for this suggestion. The manuscript already describes the differing resistance profiles across the three African countries in the Introduction. We have additionally added brief descriptions of the transmission characteristics of each site in Methods, lines 324-331.

The three sites included in this analysis represent contrasting resistance profiles: Uganda and Mozambique are East and Southern African settings where quintuple mutants predominate, while Burkina Faso is a West African setting where quadruple mutants are predominant, retaining greater susceptibility to sulfadoxine. The three study sites are all high malaria transmission settings [24]. Uganda experiences stable, perennial malaria transmission across 95% of the country [25], while Mozambique bears one of the highest malaria burdens in Eastern and Southern Africa, with transmission occurring year-round [26]. Burkina Faso, by contrast, experiences intense but markedly seasonal transmission concentrated in the rainy season [27].

“One is left wondering if the ʺsmall number of outliers” with higher levels of amodiaquine at D28 - a time where in fact, **AQ is not expected to be generally detectable** - correspond to carriers of defective CYP2C8 alleles (3). This is data easy to generate and would enrich the article, in tandem with considerations on the markedly higher pyrimethamine drug levels observed in Uganda at both D7 and D28 time points.

(3) Parikh S, Ouedraogo JB, Goldstein JA, Rosenthal PJ, Kroetz DL. Amodiaquine metabolism is impaired by common polymorphisms in CYP2C8: implications for malaria treatment in Africa. Clin Pharmacol Ther. 2007 Aug;82(2):197-203. doi: 10.1038/sj.clpt.6100122. Epub 2007 Mar 14. PMID: 17361129.”

Response:
We thank you for this insightful hypothesis. We agree that CYP2C8 polymorphisms represent a plausible explanation for the small number of children with detectable amodiaquine concentrations at day 28, as well as the markedly higher pyrimethamine concentrations observed in Uganda. Unfortunately, CYP2C8 and CYP2C19 genotyping data are not available for participants in this analysis, as host genetic analysis was not included in the original ethical clearance protocols or informed consent procedures, and generating these data retrospectively is beyond the scope of the current revision. We have acknowledged the potential pharmacogenetic basis in the Discussion lines 267-273.

For desethylamodiaquine, incomplete adherence to the second and third amodiaquine doses at sites without confirmed directly observed therapy remains the most plausible contributor to inter-site variability. CYP2C8 polymorphisms, prevalent in West African populations [20] and shown in vitro to impair amodiaquine metabolism [21], might represent an additional biological determinant. Prospective CYP genotyping in future SMC pharmacokinetic studies would help disentangle the

relative contributions of adherence and pharmacogenetic variation to inter-site differences in drug exposure.

"SP is known to have important positive effects beyond the simple protection against malaria parasites, at least in the context of IPTp, due to inherent broader anti-microbial activity. This aspect is very important for a complete evaluation of the importance of SP as a SMC partner, and should be taken in consideration in the discussion of the potential implications of the presented data."

Response:

We agree that this might represent an additional benefit of SP. Our understanding is that the non-malarial effect of SP in pregnancy is not fully demonstrated, and no studies of SMC in children (apart from one trial in sickle cell disease) has convincingly shown a non-malarial benefit. No relevant outcomes were recorded in this study to look at this potential benefit. We prefer restricting the discussion to the antimalarial benefit of SP.

"Where is the data related to the pfaat gene?"

Response:

We thank you for raising this point. Data on pfaat1 gene mutations, associated with chloroquine and lumefantrine resistance, has been added in Supplementary table S3.
